# Reliable Autism Spectrum Disorder Diagnosis for Pediatrics Using Machine Learning and Explainable AI

**DOI:** 10.3390/diagnostics14222504

**Published:** 2024-11-08

**Authors:** Insu Jeon, Minjoong Kim, Dayeong So, Eun Young Kim, Yunyoung Nam, Seungsoo Kim, Sehoon Shim, Joungmin Kim, Jihoon Moon

**Affiliations:** 1 Department of Medical Science, Soonchunhyang University, Asan 31538, Republic of Korea; jis601@sch.ac.kr; 2 Department of ICT Convergence, Soonchunhyang University, Asan 31538, Republic of Korea; wooni3804@sch.ac.kr (M.K.); sodayeong@sch.ac.kr (D.S.); eykim@sch.ac.kr (E.Y.K.); ynam@sch.ac.kr (Y.N.); 3 Department of Pediatrics, Soonchunhyang University Cheonan Hospital, Cheonan 31151, Republic of Korea; equalkss@schmc.ac.kr; 4 Department of Psychiatry, Soonchunhyang University Cheonan Hospital, Cheonan 31151, Republic of Korea; shshim@schmc.ac.kr; 5 College of Hayngsul Nanum, Soonchunhyang University, Asan 31538, Republic of Korea; 6 Department of AI and Big Data, Soonchunhyang University, Asan 31538, Republic of Korea

**Keywords:** autism spectrum disorder, clinical diagnosis, data preprocessing, healthcare analytics, machine learning, patient outcomes, personalized intervention, explainable artificial intelligence

## Abstract

**Background:** As the demand for early and accurate diagnosis of autism spectrum disorder (ASD) increases, the integration of machine learning (ML) and explainable artificial intelligence (XAI) is emerging as a critical advancement that promises to revolutionize intervention strategies by improving both accuracy and transparency. **Methods:** This paper presents a method that combines XAI techniques with a rigorous data-preprocessing pipeline to improve the accuracy and interpretability of ML-based diagnostic tools. Our preprocessing pipeline included outlier removal, missing data handling, and selecting pertinent features based on clinical expert advice. Using *R* and the *caret* package (version 6.0.94), we developed and compared several ML algorithms, validated using 10-fold cross-validation and optimized by grid search hyperparameter tuning. XAI techniques were employed to improve model transparency, offering insights into how features contribute to predictions, thereby enhancing clinician trust. **Results:** Rigorous data-preprocessing improved the models’ generalizability and real-world applicability across diverse clinical datasets, ensuring a robust performance. Neural networks and extreme gradient boosting models achieved the best performance in terms of accuracy, precision, and recall. XAI techniques demonstrated that behavioral features significantly influenced model predictions, leading to greater interpretability. **Conclusions:** This study successfully developed highly precise and interpretable ML models for ASD diagnosis, connecting advanced ML methods with practical clinical application and supporting the adoption of AI-driven diagnostic tools by healthcare professionals. This study’s findings contribute to personalized intervention strategies and early diagnostic practices, ultimately improving outcomes and quality of life for individuals with ASD.

## 1. Introduction

Autism spectrum disorder (ASD) is a complex neurodevelopmental disorder affecting approximately 1 in 54 children worldwide [1]. It is marked by ongoing challenges in social communication and interaction, along with restricted and repetitive behaviors and interests [2]. The wide range of symptoms varies greatly among individuals, making diagnosis difficult and necessitating personalized intervention strategies to effectively support each child. ASD’s profound impact extends beyond individuals to families, educational systems, and healthcare infrastructures, resulting in significant social and economic burdens [3]. Early and accurate diagnosis is crucial, as early intervention can greatly and significantly improve developmental outcomes and quality of life for children with ASD [4].

Identifying ASD is essential, as initial signs typically appear between 2 and 3 years of age, a critical period for brain development [5]. In this period, the brain’s increased plasticity makes it ideal for interventions that can positively influence developmental paths [6]. However, early symptoms are often subtle and diverse, encompassing a range of behavioral and communication issues that can be easily overlooked or misinterpreted [7]. Traditional diagnostic methods rely primarily on subjective behavioral assessments by clinicians, such as the Autism Diagnostic Observation Schedule and the Autism Diagnostic Interview—Revised (ADI-R) [8]. In addition, standardized assessment tools such as the Childhood Autism Rating Scale (CARS), the Social Responsiveness Scale (SRS), and the Autism Spectrum Quotient 10 (AQ10) are commonly used to evaluate ASD symptoms [9,10,11].

While these assessments are invaluable, their reliance on clinical expertise introduces variability and potential bias, causing inconsistencies in diagnosis [12]. Furthermore, due to the nature of ASD assessments and surveys, there may be an overwhelming representation of individuals with ASD compared to non-ASD individuals in the datasets. This imbalance can lead to decreased diagnostic accuracy when relying solely on traditional methods, as models may be biased toward overrepresented classes. In addition, shortages of trained professionals and high demand for diagnostic services exacerbate identification delays, highlighting the need for more efficient and scalable diagnostic approaches.

In recent years, artificial intelligence (AI) and machine learning (ML) has become pivotal in advancing early ASD diagnosis and intervention. ML algorithms, including support vector machines (SVMs), random forest (RF), and extreme gradient boosting (XGBoost), are highly effective in analyzing large and complex datasets [13], uncovering patterns that are undetectable by traditional methods. For example, RF and XGBoost have successfully classified ASD using behavioral and clinical data, achieving high accuracy [14,15]. These algorithms enable predictive models that improve diagnostic accuracy and support personalized intervention plans tailored to each child’s developmental [16]. In this study, we focus on survey-based behavioral and clinical data and use *R* for our analyses, prioritizing accessibility and ease of use for clinicians. By not including imaging data, we aim to develop a more streamlined and interpretable diagnostic approach, suitable for initial assessments prior to specialized testing by clinicians.

Even when AI models achieve high accuracy, it is critical to understand their decision-making processes, especially what variables they consider important beyond known factors, such as CARS, SRS, and AQ10 scores. Adopting ML in ASD diagnosis faces challenges, notably the ‘black-box’ nature of models, which lack transparency in their predictions [17]. This opacity can undermine clinician confidence and hinder AI integration in healthcare [18]. Understanding the importance of different variables, including those not typically emphasized in clinical assessments, may provide new insights and previously overlooked factors in ASD diagnosis. In addition, the absence of rigorous validation of data reliability affects the accuracy and generalizability of ML models [19]. Issues such as missing data, outliers, and biased feature selection can distort predictions [20].

To overcome these hurdles, this study incorporates explainable artificial intelligence (XAI) techniques [21], such as permutation feature importance (PFI), local interpretable model-agnostic explanations (LIMEs), and Shapley additive explanations (SHAP), to clarify ML model’s decision-making processes. These methods aim to enhance transparency and trust, ensuring that models are both accurate and interpretable [22]. This dual emphasis on performance and explainability is critical for fostering collaboration between AI systems and healthcare professionals, facilitating the integration of ML-driven diagnostic tools into clinical practices [23]. In addition, by identifying and understanding the importance of variables beyond traditional assessment tools, we can improve diagnostic criteria and support the development of more comprehensive intervention strategies.

This study underscores the paramount importance of data reliability, implementing a meticulous data-preprocessing pipeline involving outlier removal, missing data handling, and feature selection based on clinical expert input [24]. This refinement ensures that our ML models are based on high-quality data, enhancing accuracy and credibility. Our rigorous approach addresses common oversights in previous research, improving the generalizability and real-world applicability of our findings [25]. Furthermore, we provide clear guidelines to help clinicians adopt AI tools effectively in diagnostics. In addition, by using *R*, which is widely used in medicine for statistical analysis and data modeling, we aim to provide accessible and practical AI tools for clinicians without requiring extensive programming expertise. Our current study does not include imaging data, but instead focuses on survey-based assessments to streamline the diagnostic process prior to specialized testing by clinicians.

This study is guided by the following research questions:

1. What is the impact of a rigorous data-preprocessing pipeline—including outlier removal, missing data handling, and expert-driven feature selection—on the performance, robustness, and generalizability of ML models for ASD diagnosis, especially in the context of data heterogeneity and imbalance?
2. How do different ML algorithms (e.g., SVMs, RFs, XGBoost, and neural networks) compare in terms of accuracy, interpretability, and computational efficiency in diagnosing ASD using the *R* programming language and the *caret* package, and what are the trade-offs between model complexity and practical usability in clinical settings?
3. How can the integration of advanced XAI techniques (e.g., PFI, LIME, and SHAP) improve the interpretability of ML models for ASD diagnosis, and what new insights do they provide into the relative importance of different features, including those not traditionally emphasized in clinical assessments, such as CARS, SRS, and AQ10?
4. How does the development of accessible ML tools using *R* and *caret* facilitate the adoption of AI-driven diagnostic methods by clinicians, and how does this accessibility impact the effectiveness and reliability of ASD diagnosis without requiring extensive programming expertise?

This study significantly advances ASD diagnosis and prediction through several key contributions:

- We implement a careful data-preprocessing pipeline that includes outlier removal, missing data handling, and feature selection based on input from clinical experts. This rigorous approach addresses common data challenges, such as heterogeneity and imbalance, and improves the validity, robustness, and generalizability of our ML models. Our results demonstrate the critical role of data quality in the development of reliable diagnostic tools.
- We develop and rigorously evaluate several ML models—including SVMs, RFs, XGBoost, and neural networks (NNETs)—for ASD diagnosis, using *R* and the *caret* package. Using 10-fold cross-validation and grid search hyperparameter tuning, we optimize model performance and thoroughly compare their accuracy, interpretability, and computational requirements. This comprehensive analysis provides valuable insight into the trade-offs between model complexity and diagnostic efficacy.
- We integrate advanced XAI techniques, such as PFI, LIME, and SHAP, into our ML models to improve interpretability. This integration allows us to dissect the decision-making processes of complex models and uncover the relative importance of different features, including those not traditionally emphasized in clinical evaluations. These insights can inform the development of more comprehensive diagnostic criteria and personalized intervention strategies for ASD.
- Using *R* programming and the *caret* package, we create practical and accessible ML tools tailored for clinicians. Our emphasis on user-friendly implementations and model interpretability through XAI techniques ensures that these tools can be effectively integrated into clinical practice. This enables healthcare professionals to use AI-driven diagnostics without extensive programming skills, thereby increasing the accessibility and effectiveness of ASD diagnosis.

The remainder of this paper is organized as follows: Section 2 reviews existing ASD diagnosis studies and challenges. Section 3 outlines our data-collection sources, preprocessing steps, and reliability checks and describes the ML algorithms and rationale for using *R* and the *caret* package for model training and evaluation. Section 4 presents the experimental setup, performance metrics, and results of our models, including XAI application. Section 5 discusses the cross-validation results and evaluates model generalizability, noting areas for further validation and improvement. Section 6 summarizes our key findings, clinical implications, and future research directions.

## 2. Related Work

### 2.1. Machine-Learning Approaches in ASD Diagnosis

Research on ASD diagnosis highlights the vital role of early detection in enhancing children’s developmental outcomes. Table 1 summarizes previous studies’ contributions and the distinct features of our study. While earlier research demonstrated effective diagnostic tools and ML models, they lacked explainable AI techniques and rigorous data preprocessing. Our study bridges this gap by integrating advanced ML algorithms prioritizing model interpretability and data reliability, enhancing both predictive performance and clinical applicability.

Lord and Luyster’s [5] longitudinal studies demonstrated that ASD diagnoses made at age 2 remain stable through age 9, providing strong evidence for the reliability of early diagnosis. They noted that differentiating between narrowly defined autism and broader ASD categories is unnecessary owing to high variability in children’s developmental trajectories. This study highlighted the importance of early diagnosis for predicting developmental trajectories and planning appropriate interventions. McCarty and Frye [6] noted challenges in identifying behavioral abnormalities that delay diagnosis, suggesting a multi-step screening to reduce false positives. Despite the high sensitivity and specificity of M-CHAT-R/F screening tool, it has a low positive predictive value owing to ASD’s low prevalence. Bryson et al. [7] emphasized the importance of early identification and intervention in ASD, showing that early intervention can positively impact long-term developmental outcomes, enabling some children to achieve normal developmental paths. This underscores the necessity for effective early diagnostic tools and interventions.

Guthrie et al. [8] examined the stability of early ASD diagnoses and emphasized the importance of integrating clinical expertise with information from multiple sources to improve diagnostic accuracy. Their study supports a multifaceted diagnostic approach to ensure reliable and consistent ASD diagnoses. In ML for ASD, Omar et al. [12] developed a predictive model using classification and regression trees (RF-CARTs) and iterative dichotomizer 3 (RF-ID3) algorithms with the AQ-10 dataset and real-world data, achieving high accuracy across various age groups (4–11 years, 12–17 years, and 18 years and older). They also created a mobile application to make their model accessible for public use. Usta et al. [13] evaluated the performance of four ML algorithms, namely naive Bayes (NB), generalized linear model (GLM), logistic regression (LR), and decision tree (DT), in predicting ASD prognosis in 433 children, highlighting early diagnosis and intervention as key for positive outcomes. Among the models tested, the DT algorithm demonstrated the highest area under the curve (AUC) value.

Alsuliman and Al-Baity [26] developed 16 optimized ML models to improve the classification of ASD using personal and behavioral characteristics (PBCs) and gene expression (GE) data. The study applied bio-inspired algorithms, such as gray wolf optimization (GWO), flower pollination algorithm (FPA), bat algorithm (BA), and artificial bee colony (ABC), to improve feature selection and model accuracy. The GWO-SVM model achieved the highest accuracies of 99.66% (PBC) and 99.34% (GE). Ben-Sasson et al. [27] developed an ML model to predict ASD in infants using electronic health records from a national screening program. The model, validated with 3-fold cross-validation, used gradient-boosting machine (GBM) to achieve a mean AUC of 0.86 and identified developmental delay and parental concern as key predictors. Abbas et al. [28] compared the automated ML (AutoML) tools TPOT and KNIME for the detection of ASD in toddlers. TPOT achieved 85.23% accuracy, while KNIME achieved 83.89%, illustrating the benefit of feature-selection techniques for early diagnosis of ASD.

Reghunathan et al. [29] investigated different classifiers for the detection of ASD in different age groups. They used the cuckoo search algorithm for feature reduction and found key factors for ASD classification, with LR showing the highest accuracy. Bala et al. [30] developed an ML model to detect ASD across different age groups, including infants, children, adolescents, and adults. The study applied various feature selection techniques and evaluated several classifiers, with SVM performing best across all age datasets. Accuracy rates ranged from 95.87 to 99.61%. The authors used SHAP to analyze and rank the most important features to further improve classification accuracy. Batsakis et al. [31] describe an ongoing study using AI technologies to support diagnostic decision-making in clinical settings. They developed a data-driven prediction model by analyzing clinical data from past cases using an AutoML platform. Initial results are promising, but the study highlights the limitations of the available data and the need for further research to improve the model’s capabilities.

### 2.2. Advanced Techniques in ASD Diagnosis: fMRI and NLP Applications

Research on ASD has used functional magnetic resonance imaging (fMRI) and natural language processing (NLP) to study its neurological and behavioral aspects. fMRI is a technique that maps brain activity with the objective of identifying neurological differences. In contrast, NLP is a method that assesses communication patterns with the goal of enhancing our comprehension and diagnosis of ASD. Mainas et al. [32] evaluated traditional ML classifiers, such as SVM and XGBoost, and compared them with deep learning (DL) models, such as TabNet and multilayer perceptrons (MLPs), for fMRI data analysis in ASD diagnosis. They found that SVMs with radial basis function (RBF) kernels outperformed DL models, achieving an AUC of 75%, and highlighted key brain regions involved in sensory perception and attention as critical for ASD classification.

Rodrigues et al. [33] used ML and resting-state fMRI (rs-fMRI) to classify ASD severity based on brain activity. Using Autism Diagnostic Observation Schedule (ADOS) scores as a measure of severity, their study identified potential brain region biomarkers and achieved an accuracy of 73.8% in the cingulum regions, suggesting the utility of rs-fMRI data in classifying ASD severity, although they noted the need for further validation. Helmy et al. [34] reviewed the role of AI and ML in the diagnosis of ASD using various brain imaging techniques, particularly magnetic resonance imaging (MRI). The focus was on diffusion tensor imaging (DTI) and fMRI, discussing how DL has improved the early, objective, and efficient diagnosis of ASD. The paper summarized advances in AI for ASD detection and discussed future trends in the integration of AI into clinical practice.

Themistocleous et al. [35] developed an ML model using NLP to discriminate children with ASD from typically developing peers based on narrative and vocabulary skills. The model achieved 96% accuracy, with histogram-based GBM and XGBoost outperforming DTs and GBM in terms of accuracy and F1 score. This study highlights the potential of AI tools for early diagnosis of ASD, especially in underserved communities. Toki et al. [36] used ML techniques to classify ASD in children using data from a serious game specifically designed for ASD assessment. Different NNETs, including MLPs and constructed NNETs, were used, with the constructed NNET performing best, achieving 75% accuracy and 66% recall. This suggests that these techniques can increase the efficiency of ASD screening and help clinicians provide better care.

### 2.3. R’s Growing Importance in Medical Data Analysis: Paths and Perspectives

Recent studies have confirmed the growing importance of the *R* programming language in medical data analysis. Kaur and Kumari [37] used ML techniques on the Pima Indian diabetes dataset to detect patterns and risk factors. They developed predictive models using various supervised ML algorithms—linear kernel SVM, RBF kernel SVM, *k*-nearest neighbors (*k*-NN), artificial neural networks (ANNs), and multifactor dimensionality reduction (MDR)—to classify patients as diabetic or non-diabetic, demonstrating the utility of ML in early detection of diabetes. Li and Chen [38] applied classification models—DT, RF, SVM, NNET, and LR—to the Breast Cancer Coimbra Dataset (BCCD) and the Wisconsin Breast Cancer Database (WBCD). These models were evaluated using predictive accuracy, F-measure, and AUC values, with RF showing the strongest performance for breast cancer classification, highlighting its clinical relevance.

Leha et al. [39] investigated ML algorithms to predict pulmonary hypertension (PH) using echocardiographic data from 90 patients with measured pulmonary artery pressure (PAP). They applied models such as RF, lasso penalized LR, boosted classification trees, and SVMs and achieved high predictive accuracy, especially with the RF model (AUC 0.87), indicating the potential of ML to improve diagnostic support for PH. Miettinen et al. [40] investigated metabolic markers associated with chronic pain, sleep disturbance, and obesity in 193 patients undergoing pain management. Using ML and hypothesis-driven approaches, they identified key metabolites as significant for classifying patients with severe-pain phenotypes. The study found that metabolomic changes related to amino acid and methionine metabolism were associated with obesity and sleep problems, suggesting that co-occurring problems may influence chronic pain at the metabolic level.

Beunza et al. [41] compared several supervised ML algorithms, including DT, RF, SVM, NNET, and LR, to predict clinical events using data from the Framingham Heart Study. The study used two platforms, *R*-Studio and RapidMiner, and evaluated the models based on their AUC scores. NNET performed best in R-Studio (AUC = 0.71), while SVM had the highest AUC in RapidMiner (AUC = 0.75). The research highlights how ML algorithms can improve traditional regression techniques in clinical prediction. Despite these advances, previous studies often lack interpretability, posing a challenge for clinical adoption. Recognizing this gap, this study integrates XAI techniques, such as PFI, LIME, and SHAP, to improve model transparency. Improving model transparency using XAI techniques allows us to examine whether and how variables known to be important in clinical practice differ from those identified by the model.

We emphasize data reliability through a rigorous preprocessing pipeline that includes outlier removal, missing data handling, and feature selection based on input from clinical experts [31]. These enhancements increase analytical accuracy and reliability, ensuring that ML models are built on high-quality data. This approach improves the generalizability and practical applicability of research findings by addressing issues often overlooked in previous research [32]. This study provides clear guidance and best practices to help clinicians use ML tools for diagnosis in an accessible and effective manner through the *R* programming language, emphasizing improved data reliability, model interpretability, and practical use. This approach differentiates research and contributes to the development of more reliable and clinically applicable diagnostic tools for ASD.

## 3. Methods

**Figure 1:**
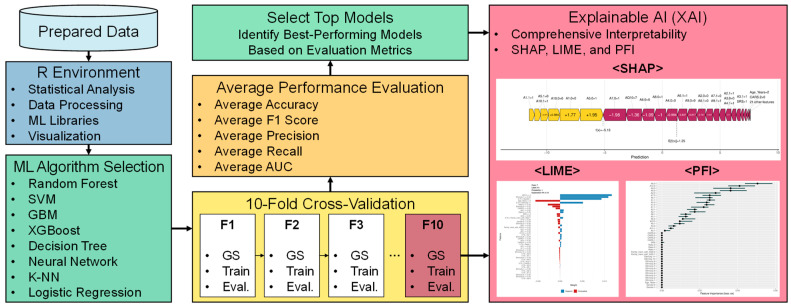
ML framework and methodology flowchart.

This section outlines the framework and methods for developing ML models for the early diagnosis of ASD. It covers the rationale for selecting specific tools and techniques, model selection and configuration, hyperparameter optimization strategies, and XAI integration to ensure model interpretability and transparency. Figure 1 illustrates the overall flow of this process, highlighting the key stages, from data preprocessing to model evaluation, with a clear emphasis on how each step contributes to the goal of accurate and interpretable ASD predictions.

### 3.1. Data Acquisition and Preparation

This study uses the ASD children trait dataset, which comprehensively captures characteristics of children with ASD, including age, gender, diagnostic criteria, and socioeconomic status. Table 2 details these variables. The dataset features identifiers for 1985 cases under *CASE_NO_PATIENT’S*. The *Social_Responsiveness_Scale* scores range from 0 to 10, with some entries missing. *Age_Years* covers ages 1 to 18, consistently captured across all entries. The *Qchat_10_Score*, assessing certain ASD traits, also ranges from 0 to 10 but includes some missing values. Binary attributes such as *Speech Delay*/*Language Disorder*, *Learning Disorder*, and *Genetic_Disorders* are clearly marked as ‘yes’ or ‘no’, without missing data, while *Depression* and *Social/Behavioral Issues* have some missing entries. Ethnicity is categorized into 11 distinct types, fully represented. This table ensures a comprehensive view of the dataset’s structure and content, aiding in the analysis of significant variables.

The data used in this study were carefully selected and restricted by clinical experts according to the following strict criteria:

1. Ensuring data completeness was a priority, and only datasets with no missing values were considered for analysis. Rather than simply excluding records with missing values, a transparent method for handling missing values was used. Missing values were handled through multiple imputations, and records were excluded from the analysis only when imputation was not feasible. For example, for the *SRS* records, data from 781 individuals—about 40% of the original 1985—were excluded because of incomplete items.
2. The analysis included only datasets that were appropriate for the age range specified by each test tool, with strict adherence to the age range specified by the test tool. For example, *QCHAT-10*, which targets infants and toddlers aged 18–24 months, required the exclusion of datasets outside this age range for reliability reasons. Similarly, *SRS* datasets that did not meet their specific age criteria were excluded. *Age* criteria for each instrument were determined based on the relevant literature in order to maintain validity and reliability.
3. To improve the reliability of the test responses, cases categorized as ‘other’, ‘school and NGO’, or ‘self’ were excluded from the analysis, as they were considered less reliable. This exclusion criterion was based on previous studies indicating that results can vary significantly depending on the respondent, making them statistically unreliable.
4. Any datasets that were likely to introduce prediction error or statistical bias were omitted during the analysis phase. For example, cases in which the responses to items A1–A10 were all zeros were excluded because they showed insufficient variability to effectively predict autism. This exclusion was considered justified because previous analyses confirmed a low correlation with autism prediction.

**Figure 2:**
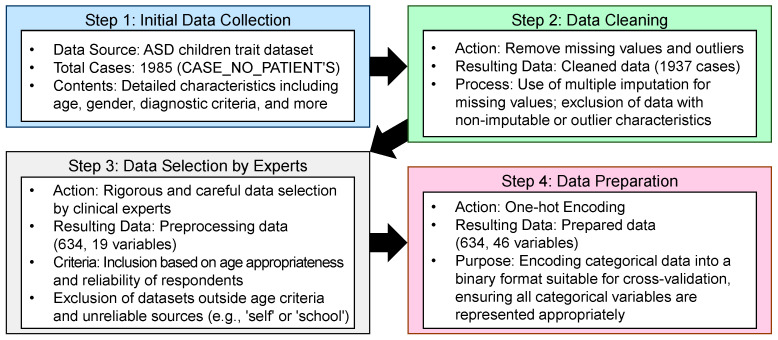
Flowchart of data selection and analysis process.

To identify the most effective ML model for ASD diagnosis, we first imported the dataset using *readxl* [42] and performed a thorough preprocessing to ensure compatibility with the different models. This preprocessing allowed us to test a variety of algorithms, each chosen for their ability to handle complex, high-dimensional data and provide insight into feature importance. Through these strict data-selection criteria, we ensured the consistency and reliability of the data, thereby guaranteeing the accuracy and validity of the analysis results. Figure 2 briefly illustrates the data-processing steps used to ensure accurate analysis.

In our study, the dataset underwent a comprehensive transformation process to ensure its suitability for analyzing ASD traits. Key transformations included the following:

- Gender transformation: The original *Sex* variable (‘F’ for female; ‘M’ for male) was converted to numerical values (males as 1; females as 2) for easier analysis.
- Age preservation: The *Age_Years* variable remained unchanged to ensure reliable age-related analysis.
- Ethnicity recoding: The *Ethnicity* variable, initially comprising various ethnic descriptors, was recoded into numeric identifiers (e.g., Asian as 1, Black as 2, and Hispanic as 3) for standardized modeling inputs.
- Family history of ASD: The *Family_mem_with_ASD* variable, which originally documented responses as ‘yes’ or ‘no’, was transformed into a binary format (1 for ‘yes’; 2 for ‘no’) to simplify familial ASD analysis.
- Rater categorization: The *Who_completed_the_test* variable was reclassified into *Rater*, encoding family members as 1 and healthcare professionals as 2.
- ASD traits: The *ASD_traits* variable retained its binary format, with ‘yes’ as 1 and ‘no’ as 2 for analysis.
- Social Responsiveness Scale (SRS): Scores from 1 to 10 were kept to measure social-responsiveness severity.
- Autism diagnostic scores: The *Childhood Autism Rating Scale* variable consolidated various diagnostic metrics into a single score, reflecting autism severity, categorizing symptoms from ‘nothing’ to ‘severe’, thereby standardizing ASD severity assessments.
- Autism Quotient Score: The *Qchat_10_Score* variable was renamed to AQ10 in the cleaned dataset. This score quantifies autism severity on a scale from 1 to 10, standardizing diagnostic outcomes across different assessments.
- Other ASD-relevant variables (A1–A10): ASD-relevant variables, labeled from A1 to A10, reflect diagnostic criteria or behavioral observations. These variables were standardized to ensure uniformity across datasets, enhancing their analytical use.

Meticulous data transformations enhanced analytical clarity and aligned the dataset with established standards, ensuring robust ASD-related analyses. Data reliability, crucial in social science research, was verified through statistical validation and expert review. Clinical experts identified inconsistencies, increasing dataset reliability by removing erroneous data. After preprocessing and reliability checks, the dataset was refined to 634 samples and 19 variables from 1985 samples and 28 variables, focusing on variables essential for ASD diagnosis. This refined dataset optimized predictive performance for ML models. Table 3 classifies each variable, detailing ranges and categories for clarity, while the preprocessed dataset used for our experiments, along with brief descriptions of the values and characteristics of each variable, is available in Table S1 (ASD_Preprocessed_Dataset.xlsx).

### 3.2. R: A Robust Environment for Statistical Analysis and ML

The *R* programming language was chosen for ASD diagnostic models because of its strengths in statistical analysis, data manipulation, and ML [43]. Its user-friendly libraries facilitate complex analyses for non-experts, thus assisting healthcare professionals. *R* excels in data processing, with packages such as *dplyr* and *data.table*, which are essential for preparing datasets for model training [44,45]. Visualization tools such as *ggplot2* and *lattice* libraries [46,47] have improved data interpretation for data scientists and clinicians. The *caret* package provided a unified interface for implementing a wide variety of ML algorithms [48], streamlining training and evaluation. In addition, specialized packages optimized for specific ML techniques, such as *xgboost*, *randomForest*, and *nnet*, integrate seamlessly with *R*, further enhancing model versatility and performance. *R* supports automated cross-validation and hyperparameter tuning, ensuring efficient model optimization and reproducibility. In summary, *R*’s comprehensive suite of statistical and ML tools, combined with its power and flexibility, made it an ideal platform for both data preprocessing and model development in this study.

### 3.3. Selection and Configuration of ML Algorithms

To identify the most effective ML model for ASD diagnosis, a diverse set of algorithms was tested for their ability to handle complex, high-dimensional data and provide insights into feature importance. The evaluated algorithms include the following:

- Random forest (RF) [49,50]: Utilized the *randomForest* package. This ensemble-learning method improves prediction performance and reduces overfitting by using multiple DTs. Equation (1) shows that the RF model predicts the output based on the majority vote from multiple DTs, thus improving accuracy and robustness.

*y_hat_* = majority_vote(*T*_1_(*x*), *T*_2_(*x*), ..., *T_n_*(*x*)). (1)

- Support vector machine (SVM) [51,52]: Implemented using the *e1071* package. SVM is effective in high-dimensional spaces and captures complex patterns by finding the optimal separating hyperplane. Equation (2) determines the classification of SVM by computing a hyperplane in high-dimensional space that best separates the classes. *alpha_i_*, *y_i_*, and *K* represent support vectors, labels, and the kernel function, respectively, enhancing SVM’s ability to model complex relationships.

*f*(*x*) = sign(sum(*alpha_i_* × *y_i_* × *K*(*x_i_*, *x*) + *b*)). (2)

- Gradient-boosting machine (GBM) [53,54]: Operated using the *gbm* package. GBM increases model accuracy by sequentially correcting errors from previous models, effectively handling complex data relationships. Equation (3) updates the prediction model by incrementally improving errors, where *h_m_*(*x*) is the improvement term, and *v* is a scaling factor that helps fine-tune the correction.

*F_m_*(*x*) = *F*_(*m* − 1)_(*x*) + *v* × *h_m_*(*x*). (3)

- XGBoost [55,56]: Configured using the *xgboost* package. It is an optimized version of GBM that includes additional regularization and parallel processing to improve speed and performance. Equation (4) represents XGBoost’s loss computation, which not only focuses on reducing the prediction error (*l*(*y_i_*, *y_hat_i_*)), but also includes a regularization term (*Omega*) to prevent overfitting.

*L*(*theta*) = sum(*l*(*y_i_*, *y_hat_i_*) + sum(*Omega*(*f_k_*))). (4)

- C5.0 Decision Tree [57,58]: Facilitated by the *C50* package. C5.0 simplifies complex tree structures to improve interpretability and predictive accuracy. Equation (5) calculates the information gain from using attribute, *A*, to split set, *S*, which helps decide the best splits to improve tree accuracy and simplicity.

*Gain*(*S*, *A*) = *Entropy*(*S*) − sum((|*S_v_*|/|*S*|) × *Entropy*(*S_v_*)). (5)

- Neural network (NNET) [59,60]: Built with the *nnet* package. NNET models complex nonlinear relationships and is powerful for pattern recognition, although it is resource intensive. Equation (6) represents the activation of a neuron, where sigma is the activation function, *w_ij_* is the weight, *x_i_* is the input, and *b_j_* is the bias, illustrating the computational process of the neuron.

*a_j_* = sigma(sum(*w_ij_* × *x_i_* + *b_j_*)). (6)

- *k*-nearest neighbors (*k*-NN) [61,62]: Implemented through the *class* package. *k*-NN classifies instances based on the proximity of the *k* nearest training data points. Equation (7) averages the labels *yi* of the *k*-nearest neighbors to predict the class, demonstrating *k*-NN’s reliance on local data similarity.

*y_hat_* = (1/*k*) × sum(*yi*). (7)

- Logistic regression [63,64]: Used the *glmnet* package. This model assumes a linear relationship between variables and outcomes, effectively modeling binary data. This logistic function (Equation (8)) calculates the probability that the outcome is 1 based on the linear combination of the input features, *x*, weighted by *w*, plus a bias term, *b*, illustrating a simple yet effective classification approach.

*P*(*y* = 1|*x*) = 1/(1 + exp(−(*w* × *x* + *b*))). (8)

Each model is selected to provide a comprehensive comparison that addresses both linear and nonlinear relationships and focuses on accuracy, reliability, and interpretability in ASD diagnosis.

### 3.4. Hyperparameter Optimization and Grid Search Using Caret

Hyperparameter optimization is critical for enhancing ML model performance [65], significantly affecting accuracy and generalizability. This study utilized a grid search approach to systematically explore the hyperparameter space for each algorithm, ensuring optimal performance [66]. The optimization process was integrated with ten-fold cross-validation to validate model performance and prevent overfitting.

#### 3.4.1. The Caret Package: An Overview

The *caret* package in *R* is a comprehensive toolkit that simplifies training, tuning, and evaluating ML models [48]. It provides a consistent interface to a wide array of ML algorithms, facilitating the implementation of standardized workflows for model development. The key reasons for utilizing *caret* in this study are as follows:

- Unified interface for diverse models: *Caret* offers a consistent set of functions for training and evaluating different algorithms, streamlining workflows and simplifying model comparison.
- Automated cross-validation and resampling [67]: *Caret* automates cross-validation and resampling to assess model performance and ensure generalization to unseen data, reducing human error and enhancing reproducibility.
- Efficient hyperparameter tuning: A standout feature of *caret* is its ability to perform hyperparameter tuning through grid search and other optimization techniques. By automating hyperparameter space exploration, it boosts model performance with minimal manual effort.
- Feature engineering and preprocessing integration: *Caret* seamlessly integrates feature-engineering and data-preprocessing steps into the modeling pipeline, ensuring consistent data transformations and fair model comparisons.
- Performance metrics and model comparison: *Caret* provides built-in functions for calculating various performance metrics and supports side-by-side model comparisons to identify best-performing algorithms.
- Reproducibility and documentation: By encapsulating the entire modeling process within a single framework, *caret* enhances the reproducibility and allows comprehensive documentation for easy replication and validation.

#### 3.4.2. Implementation of Hyperparameter Optimization with Caret

1. Setting up cross-validation and train control: A ten-fold cross-validation strategy was established using *trainControl* within *caret*. This setup involved the following:
  - Method: Cross-validation to partition the data into training and testing sets.
  - Number: Ten for ten-fold cross-validation, ensuring that each model was evaluated on ten different data subsets.
  - Search: Grid to perform grid search hyperparameter tuning.
  - Class Probabilities: Enabled (*TRUE*) to allow for probability-based metrics.
  - Summary Function: *multiClassSummary* to calculate various performance metrics suitable for multi-class classification problems.
2. Defining hyperparameter grids for each model: Specific hyperparameter grids were developed for each algorithm to explore different configurations. The following are examples:
  - RF: Tuned the *mtry* parameter, representing the number of variables considered at each split.
  - SVM: Adjusted the *C* (cost) and *sigma* parameters for the radial basis function kernel.
  - GBM: Modified parameters such as *n.trees*, *interaction.depth*, *shrinkage*, and *n.minobsinnode*.
  - XGBoost: Focused on *nrounds*, *max_depth*, *eta*, *gamma*, *colsample_bytree*, *min_child_weight*, and *subsample*.
  - C5.0 Decision Tree: Tuned *trials*, *model*, and *winnow*.
  - NNET: Tuned *size* (number of neurons in hidden layers) and *decay* (weight decay rate).
  - *k*-*nearest* neighbors (*k*-NN): Tuned the number of neighbors (*k*).
  - Logistic *regression* (GLMNET): Tuned the *lambda* parameter for regularization.
3. Training models with *caret*: Each ML model was trained using the train function from *caret*, which seamlessly integrated the defined hyperparameter grids and cross-validation strategy. This consistent approach was applied across all models, ensuring a standardized training process.
4. Evaluating model performance: After training, each model was evaluated using resampling techniques provided by *caret*. Metrics such as accuracy, F1 score, precision, recall, and AUC were calculated to assess their effectiveness [48].
5. Selecting top-performing models: Based on the evaluation metrics, the top-performing models were identified for further analysis and interpretation. This selection was crucial for focusing subsequent efforts on models demonstrating the highest potential for accurate and reliable ASD diagnosis.

#### 3.4.3. Advantages of Using Caret for Hyperparameter Optimization

The use of the *caret* package in this study provided numerous advantages that significantly enhanced the efficiency and effectiveness of hyperparameter optimization:

- Streamlined workflow: *Caret’s* unified interface enabled a seamless workflow for data preprocessing, model training, hyperparameter tuning, and evaluation within a single framework. This reduced the need for switching between different packages and functions, reducing complexity and potential errors.
- Comprehensive model tuning: Through grid search, *caret* enabled exhaustive exploration of hyperparameter spaces, optimizing configurations for each model. This is crucial for enhancing performance and adapting models to ASD dataset characteristics.
- Consistency across models: By providing a standardized approach, *caret* ensured consistency in evaluating and comparing algorithms, vital for unbiased and accurate model assessments.
- Efficiency and speed: *Caret’s* ability to parallelize computations accelerated tuning, especially beneficial for large datasets and complex models requiring extensive tuning.
- Robust evaluation metrics: *Caret* offers a wide range of performance metrics and supports multi-class classification evaluations, essential for accurately assessing models in the context of ASD diagnosis.
- Reproducibility and documentation: *Caret* facilitates documenting and reproducing the modeling process, ensuring other researchers can replicate procedures and validate findings and thus enhancing credibility.
- Flexibility and extensibility: *Caret* is highly flexible, allowing customization to specific needs. Its extensibility helps integrate new models and techniques, aligning with the latest ML advancements.

In summary, *caret* was instrumental in efficiently managing hyperparameter optimization, ensuring meticulous model tuning for optimal performance. Its features and ability to standardize workflows rendered it an invaluable tool in developing reliable diagnostic models for ASD.

### 3.5. Enhancing Model Transparency with XAI

In healthcare, especially when diagnosing complex conditions like ASD, the transparency and interpretability of ML are crucial. Clinicians must not only trust the accuracy of these models but also understand the rationale behind their predictions to confidently integrate AI tools into clinical practice. This transparency is particularly important in high-stakes domains like healthcare, where decisions significantly impact patient outcomes. Without clear insights into model predictions, clinicians may hesitate to rely on AI-generated diagnoses. To address the ‘black box’ nature of sophisticated algorithms, such as NNETs and ensemble methods like XGBoost, this study incorporated various XAI techniques [68]. XAI provides tools to clarify the internal workings of these models, offering explanations understandable to clinicians and stakeholders [69]. By leveraging these techniques, we transformed ML models from opaque decision-makers into transparent and interpretable tools, enhancing their trustworthiness in clinical settings.

In this study, we employed three main XAI techniques: PFI [70], LIME [71], and SHAP [72]. Each method contributed to our understanding of the models’ prediction processes. Below, we explain each technique and how it was used to enhance model interpretability. For clarity and transparency in our ML model analysis for ASD diagnosis, we used the following *R* packages to implement specific XAI techniques alongside our best models:

- *iml* package: Used for SHAP scores and PFI, which evaluates the impact of each feature on model predictions and helps identify the most important predictors.
- *lime* package: Applied for LIME, which provides local explanations that help clarify why certain predictions were made by the model.

These tools were integrated after selecting the best performing models based on accuracy, ensuring that our explanations are relevant and directly applicable to the most effective models.

#### 3.5.1. PFI

PFI is a model-agnostic technique used to measure feature importance by observing performance degradation when feature values are randomly shuffled. If shuffling a feature causes a notable accuracy drop, the feature is crucial for predictions; if not, it likely has little impact. We applied PFI to identify key variables in predicting ASD. By shuffling values like ‘*Frequency of Eye Contact*’ or ‘*Family History of ASD*’, we gauged accuracy changes, ranking features by importance (Equation (9)).

Delta Accuracy = Accuracy*_original_* − Accuracy*_permuted_*. (9)

Our study found behavior-related variables, such as communication patterns and repetitive actions, to be more influential than demographic factors, like age or gender. This methodology ensures a comprehensive understanding of the impact of each feature on the accuracy of the model, thereby enhancing the interpretability of our results.

#### 3.5.2. LIME

While PFI focuses solely on global feature importance, LIME focuses on generating localized explanations for individual predictions. LIME creates an interpretable surrogate model, typically a simpler, linear model that approximates the complex model’s behavior near a particular prediction. This is especially useful for understanding why a model classified a specific patient as having ASD (Equation (10)).

*y* = *wx* + *b*. (10)

In clinical practice, LIME provides transparent explanations for each diagnosis, helping clinicians trust the model outputs. For example, if a model identifies a patient with ASD, LIME can show which features, like limited eye contact or repetitive behaviors, were most influential. Such case-by-case analyses allow clinicians to cross-check the model reasoning with their own clinical judgment, building confidence in AI-driven diagnoses.

#### 3.5.3. SHAP

SHAP, rooted in cooperative game theory, provides a unified approach to measure each feature’s contribution to a model’s prediction. By calculating ‘SHAP values’, it assigns an importance score to each feature, reflecting its impact on the prediction for a specific data point. This is especially useful for understanding how variables like behavioral traits, family history, and demographic information affect the likelihood of an ASD diagnosis. A key advantage of SHAP is its consistent application across diverse ML models. Whether applied to tree-based models like RF and XGBoost or complex models like NNETs, SHAP offers a consistent framework for understanding feature importance. The contribution of each feature *i* to a prediction can be expressed quantitatively by Equation (11):

Phi(*i*) = sum((*v*(*S* U {*i*}) − *v*(*S*))). (11)

In this study, we used SHAP to analyze key variables, such as behavioral indicators (e.g., repetitive actions or communication challenges), and their effect on the model’s ASD classification decisions. SHAP provided both global explanations, highlighting the most influential features across the dataset, and local explanations, offering insights into individual predictions.

#### 3.5.4. Integrating XAI Techniques for Comprehensive Interpretability

The combination of PFI, LIME, and SHAP provided a comprehensive set of explanations for our ML models. PFI ranked features based on their impact on performance, providing an initial global understanding. LIME then added local insights, helping clinicians understand features that are important for specific diagnoses. Finally, SHAP provided both global and local explanations, deepening the understanding of model behavior at multiple levels. Together, these techniques transformed complex models into interpretable tools that clinicians could trust. This transparency is critical to the adoption of AI-based diagnostic tools in healthcare, ensuring that predictions are consistent with clinical reasoning and real-world observations. In addition, XAI methods emphasized the importance of behavioral factors in diagnosing ASD, highlighting the need to prioritize these features in clinical practice.

## 4. Results

This section analyzes the experimental results from evaluating multiple ML models for diagnosing ASD. We set the *tuneLength* parameter to 10 to optimize the hyperparameters of each ML model through a comprehensive grid search. The models were assessed using various performance metrics to compare their predictive capabilities in a robust way. XAI techniques were also employed to enhance model transparency and interpretability.

### 4.1. Model Performance Evaluation

Each model’s performance was evaluated using several key metrics: accuracy, F1 score, area under the precision–recall curve (prAUC), precision, and recall. These metrics offer a comprehensive understanding of each model’s effectiveness in diagnosing ASD. Accuracy, reflecting the proportion of correct predictions, is critical for understanding overall model success. The F1 score balances precision and recall, particularly valuable when class imbalance could skew accuracy calculations. The prAUC metric was vital in this study, as it evaluates the model ability to distinguish true positives from false positives, essential for early and accurate ASD detection.

Table 4 summarizes the overall performance metrics of the machine-learning models evaluated in our study for diagnosing ASD. The NNET and XGBoost models achieved the highest accuracy scores of 1 and 0.9984, respectively. These models also excelled in F1 score and prAUC, highlighting their effectiveness in discriminating between ASD and non-ASD cases. Such high performance suggests that these models are highly reliable for our diagnostic purposes. To statistically validate the performance differences among the models and to select the best one for our work, we performed the Wilcoxon signed-rank test and the Friedman test based on the fold-wise performance metrics obtained from the 10-fold cross-validation. The detailed performance metrics for each fold are presented in Appendix A (Table A1, Table A2, Table A3, Table A4, Table A5, Table A6, Table A7, Table A8, Table A9 and Table A10).

The Wilcoxon signed-rank test is a nonparametric statistical method used to compare two related samples to determine whether their population mean ranks differ [73,74]. In this context, it was used to compare the NNET model with each of the other models across all folds, testing the null hypothesis that there is no significant difference in performance between NNET and the compared model. Table 5 presents the *p*-values from the Wilcoxon signed-rank test comparing the NNET model to each of the other models on various performance metrics. A *p*-value less than 0.05 indicates a statistically significant difference in favor of the NNET model.

The results show that NNET significantly outperforms RF, C5.0, and *k*-NN for all performance metrics, as evidenced by *p*-values well below 0.05. When compared to SVM, GBM, XGBoost, and LR, the differences are not statistically significant for certain metrics, suggesting that these models perform comparably to NNET in some aspects. The Friedman test is another nonparametric test used to detect differences in treatments across multiple trials, especially when comparing more than two groups [74,75]. It assesses whether there are significant differences in the performance of all models across all folds. Table 6 shows the results of the Friedman test for each performance metric. The extremely low *p*-values (all less than 0.05) indicate that there are statistically significant differences between the models for each metric evaluated.

These statistical analyses reinforce the results presented in Table 4, confirming that the NNET model consistently outperforms other models, especially RF, C5.0, and *k*-NN. The significant *p*-values from the Wilcoxon signed-rank test highlight the robustness of NNET’s performance across multiple metrics. In addition, the Friedman test indicates overall significant differences among all models tested, further justifying the selection of NNET as the most effective model for our work. By demonstrating statistically significant improvements in key performance metrics, the NNET model proves to be highly effective in discriminating between ASD and non-ASD cases.

**Figure 3:**
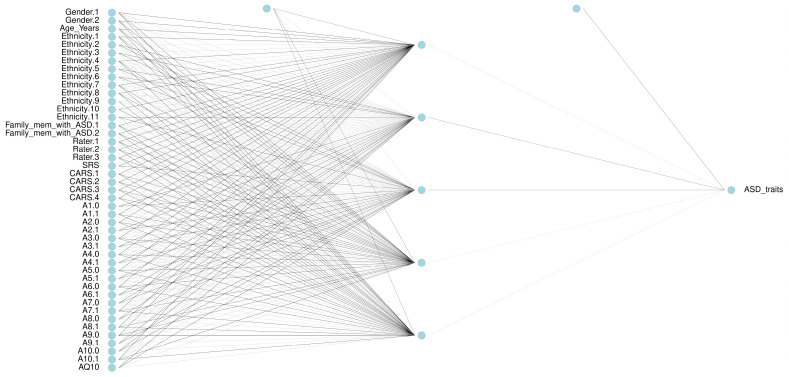
Neural network (NNET) architecture for ASD diagnosis.

To achieve these optimal performance metrics, different neural network architectures were systematically tested, resulting in the architecture shown in Table 7 and Figure 3. Through a grid search on the NNET model, we found that a hidden layer size of 5 neurons and a decay of 0.1 provided the best performance and learning speed. The results for each combination of size and decay are detailed in Table 7. In addition, Figure 3 illustrates the architecture of the neural network used, which consists of an input layer with 45 nodes, a hidden layer with 5 nodes, and an output layer with 1 node.

GBM also demonstrated strong performance, achieving similarly high accuracy and precision, marking it another top-performing model. Conversely, *k*-NNs and C5.0 DTs demonstrated lower performance, particularly in prAUC and F1 scores, indicating that they may not be as suitable for ASD diagnosis within this dataset. Despite these differences, all models contributed valuable insights into handling ASD data complexities. To further understand the varying performance of *k*-NN, we analyzed how different *k*-values affect its accuracy, which helped to identify the optimal configuration for ASD diagnosis.

Table 8 provides a detailed analysis of the performance metrics of the *k*-NN model, including the accuracy, F1 score, and prAUC, for different *k*-values. The analysis shows that *k* = 7 achieves the best balance of sensitivity and specificity, establishing it as the optimal setting for the *k*-NN model in this study. Although *k* = 7 shows the best performance among the *k*-NN settings, it inherently lacks the robust predictive power of models such as XGBoost, NNET, and GBM, primarily due to its simpler, proximity-based algorithm, which may not capture complex patterns as effectively.

### 4.2. Results of XAI

In addition to evaluating performance metrics, we applied XAI techniques—specifically SHAP, LIME, and PFI—to our top-performing models, including GBM, XGBoost, and NNET, to improve our understanding of their decision-making processes. These techniques are used to interpret and explain the predictions made by these models. By using methods such as SHAP scores and variable importance scores provided by LIME and PFI, we can determine which variables significantly influence predictions. A higher value assigned to a variable indicates that it is more important to the model and therefore plays a critical role in the outcome of the predictions. This approach ensures a comprehensive analysis, providing a deeper insight into how these models process and analyze data and eliminating any ambiguity about their function.

**Figure 4:**
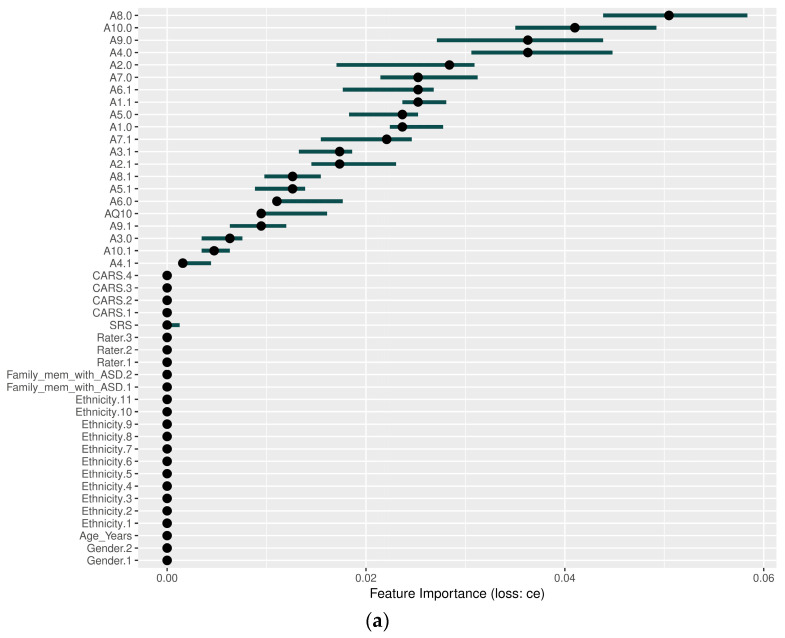

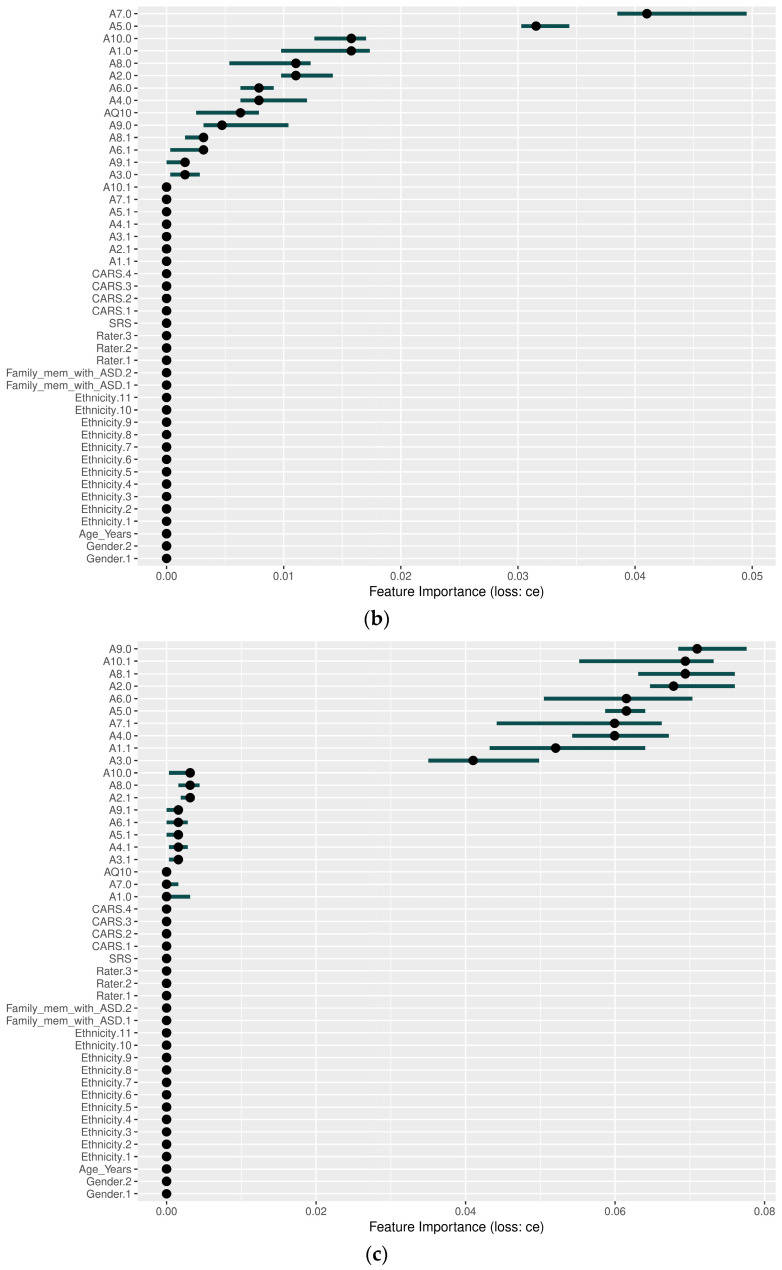
Permutation feature importance (PFI) results: (**a**) GBM, (**b**) XGBoost, and (**c**) neural network.

Figure 4 shows the results of the PFI analysis, where the *x*-axis represents the ‘Feature Importance (loss: ce)’. This figure illustrates the extent to which the cross-entropy loss of the model is amplified when the values of one feature are randomly shuffled while the values of other features remain unchanged. A higher value on the *x*-axis indicates that a given feature has a greater impact on the model’s predictions. The *y*-axis displays the features in descending order of importance, with the most critical features listed first. The dots represent the estimated importance of each feature, while the horizontal lines show the variability, indicating the uncertainty in the importance of the feature across different permutations. Detailed explanations of the GBM, XGBoost, and NNET models are provided below:

- In the GBM model, as shown in Figure 4a, behavioral characteristics such as *A8.0*, *A10.0*, *A9.0*, and *A4.0* were the most influential, while demographic variables such as gender and ethnicity had little impact. *A8.0* emerged as the top variable, while other behavioral indicators played key roles.
- Similarly, as shown in Figure 4b, XGBoost highlighted significant behavioral variables such as *A7.0* and *A5.0*, while clinical and demographic variables such as *CARS*, *Family_mem_with_ASD*, and *gender* had little impact on model predictions, reinforcing the emphasis on behavioral characteristics.
- In the NNET model, as shown in Figure 4c, behavioral variables such as *A9.0*, *A10.1*, *A8.1*, and *A2.0* significantly influenced predictions, while traditional clinical variables such as *CARS*, *SRS*, and *Family_mem_with_ASD* were rarely used. Variables *A10.1* and *A6.0* contributed with a wide range of uncertainty, suggesting context-dependent importance.
- Behavioral characteristics are consistently shown to be highly influential in all models, while demographic variables have a comparatively small impact. The longer bars indicate greater uncertainty in the importance of some characteristics, but overall, the results highlight the critical role of behavioral characteristics in driving model predictions.

**Figure 5:**
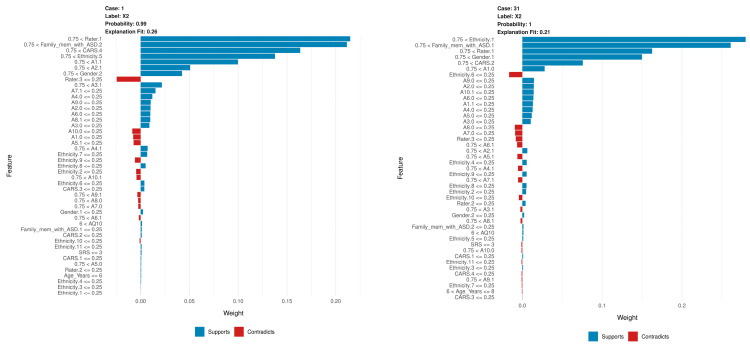

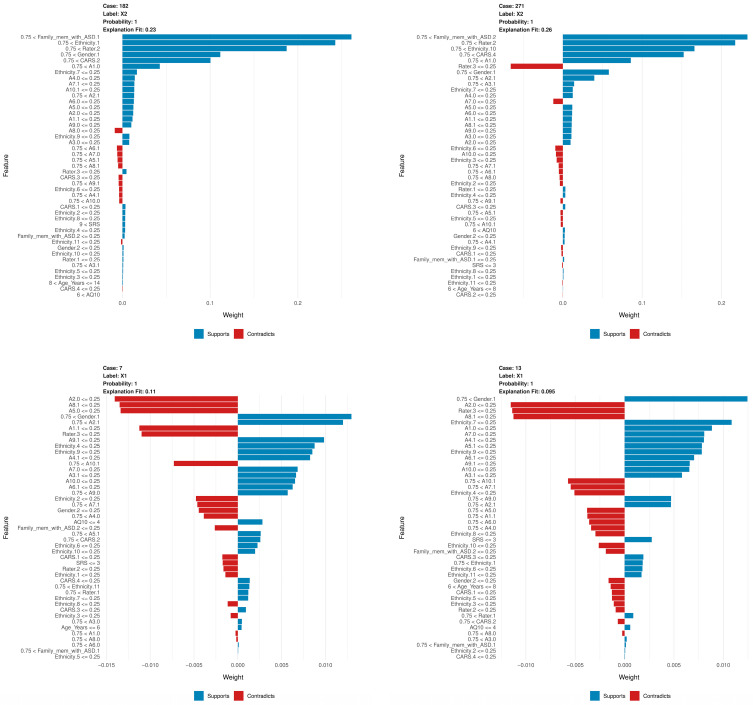
Local interpretable model-agnostic explanation (LIME) results in six different cases, using the NNET model.

**Figure 6:**
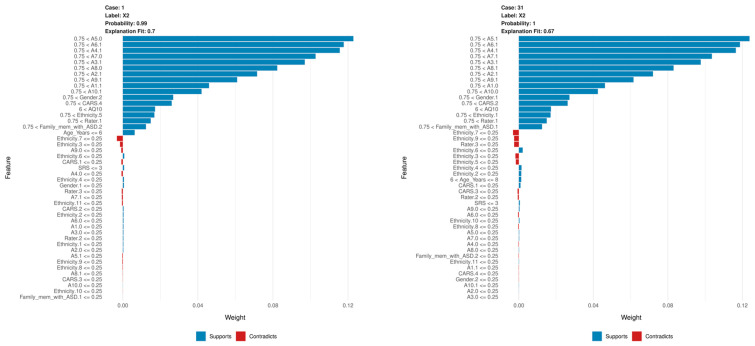

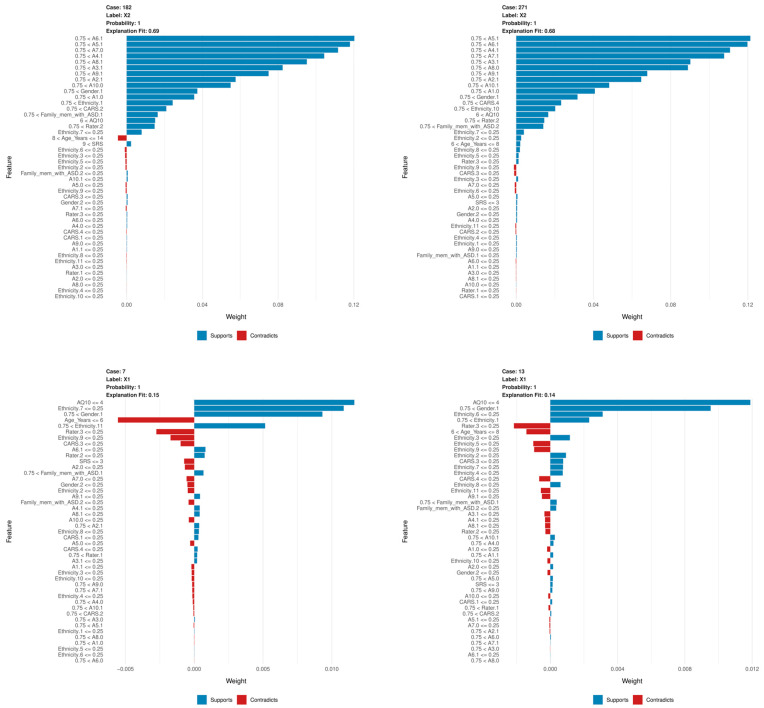
LIME results in six different cases using the XGBoost model.

LIME provides localized explanations for individual diagnoses, showing probability and explanation fit. This helps to assess the reliability of the model predictions. When evaluating NNET and XGBoost with LIME, similar to the PFI and SHAP results, variables *A1* through *A10* have a high weight and contribute to the prediction of variables. In general, the likelihood and explanation fit were high, but occasionally they were low, and the feature contributions differed from SHAP and PFI, as shown in Figure 5 and Figure 6.

In this context, the term ‘case’ is used to denote a specific observation derived from the dataset under consideration. The assignment of a case number represents a specific instance. For example, ‘Case 1’ denotes the first observation. The ‘label’ denotes the class predicted by the model, where ‘label X2’ indicates that the model assigned this instance to category *X2*. The ‘probability’ value represents the degree of confidence the model has in its prediction. Thus, a probability of 0.99 means that the model is 99% certain that the case belongs to the class *X2*. The ‘explanation fit’ metric quantifies the extent to which LIME’s explanation matches the model’s behavior for a given case. A higher value, approaching 1, indicates that LIME has a satisfactory fit to the model’s decision.

Figure 5 shows the LIME for six cases using the NNET model. Below is a summary of each case, highlighting the main factors that support or contradict the model’s predictions:

- In Case 1, the model predicted label *X2* with an accuracy of 0.99 and an explanation fit of 0.26, indicating a moderate level of interpretability. The LIME explanation shows that the most influential features in supporting the prediction are *Rater*, *Family_mem_with_ASD*, and *CARS*. These features played a significant role in the model’s decision, with *Rater* being the most significant contributor. However, characteristics *A10* and *A1* slightly contradict the prediction, although their influence is less significant compared to the supporting factors.
- In Case 31, the model predicted label *X2* with an accuracy of 1 and an explanation fit of 0.21, showing lower interpretability. The LIME results indicate that *Ethnicity* was the most influential feature, followed by *Family_mem_with_ASD*, *Rater*, and *Gender*. The combination of these features primarily drove the model’s prediction toward label *X2*, with *Ethnicity* being particularly influential. On the other hand, some ethnicity variables, as well as lower scores on traits *A8* and *A7*, contradict the prediction, although they have relatively small effects.
- In Case 182, the model also predicted Label X2 with an accuracy of 1 and an explanation fit of 0.23, which is relatively low. According to the LIME results, *Family_mem_with_ASD*, *Ethnicity*, and *Rater* were the main contributors to this prediction. The high importance of *Family_mem_with_ASD* and *Ethnicity* strongly biased the model toward the *X2* label. However, some ethnicity traits and lower scores on trait *A8* act as opposing factors, but their influence is comparatively weaker.
- In Case 271, the model predicted label *X2* with an accuracy of 1 and an explanation fit of 0.26, similar to Case 182. The LIME explanation shows that *Family_mem_with_ASD*, *Ethnicity*, and *Rater* were again the most influential features. This consistency across cases suggests that these features are critical to the model’s decision to predict label *X2*. Conversely, certain ethnicity characteristics and a lower score on trait *A7* contradict the model’s decision, although their influence is relatively small.
- In Case 7, the model predicted label *X1* with an accuracy of 1 and a relatively low explanation fit of 0.11. Here, *Gender* was a positive contributor to the prediction of X1, while *A2* and *Rater* were negative contributors, making the interpretation more complex due to the mixed influences of these features.
- In Case 13, the model, again, predicted label *X1* with an accuracy of 1 and an explanation fit of 0.095, indicating low interpretability. In this case, *Gender* contributed positively to the prediction, but *A2* and *Rater* had negative effects. This combination of opposing influences presents additional challenges in interpreting the model’s decision for label *X1*.

Figure 6 shows the LIME for six cases using the XGBoost model. Below is a summary of each case, highlighting the main factors that support or contradict the model’s predictions:

- For Case 1, the label is X2, the probability is 0.99, and the explanation fit is 0.7. The LIME explanation shows that several features, such as A5, A6, and A4, contribute significantly to the prediction of class X2. These features have the highest positive weights, supporting the model’s decision. Although some smaller features, shown in red, contradict the prediction, their impact is negligible. The model has a very high confidence of 99%, and the explanation fit is relatively strong at 0.7, indicating that the LIME explanation for this case is in good agreement with the model’s behavior.
- For Case 31, the label is also X2, with a probability of 1 and an explanation fit of 0.67. As in Case 1, the top features supporting classification into X2 include A5, A6, and A4. Although a few features related to ethnicity and CARS scores have a small negative impact on the prediction, their contribution is minimal compared to the top supporting features. The model’s confidence is perfect, with a probability of 100%, although the explanation fit is slightly lower than in Case 1, at 0.67.
- In Case 182, the label remains X2 with a probability of 1 and an explanation fit of 0.69. The same key features as in the previous cases, such as A5, A6, and A7, play a large role in supporting the model’s decision. There are small negative contributions from features such as ethnicity, but these are overshadowed by the stronger supporting features. The model’s confidence is absolute, with a probability of 1, and the explanation fit is moderate, at 0.69, indicating reasonable agreement between LIME’s explanation and the model’s prediction.
- For Case 271, the label is X2, the probability is 1, and the explanation fit is 0.68. As with the other cases predicting X2, features A5, A6, and A4 contribute the most to the prediction. While some features related to ethnicity have a small negative influence, the overall prediction is overwhelmingly supported by the positively contributing features. The model again shows perfect confidence with a probability of 1, and the explanation fit is close to 0.7, indicating a high level of interpretability for this case.
- In contrast, Case 7 predicts label X1 with a probability of 1 and an explanation fit of 0.15. The low explanation fit of 0.15 suggests that LIME’s explanation does not fit well with the model’s decision process for this case. Several features, such as AQ10 and Ethnicity, contribute positively to the prediction of class X1, but there are also significant negative contributions from features such as Age_Years and Rater. This mix of support and contradiction makes the explanation more ambiguous and difficult to interpret compared to the earlier X2 cases.
- Finally, for Case 13, the model also predicts class X1 with a probability of 1 and an explanation fit of 0.14. Like Case 7, this case has a low explanation fit, which makes it more difficult to interpret the model’s decision with confidence. The top features, such as AQ10 and Gender, provide strong positive support for the prediction of X1. However, many other features, particularly those related to Rater and Age_Years, provide negative contributions, complicating the overall explanation. This case, similar to Case 7, is less straightforward compared to the higher explanation fits seen in the X2 cases.

The LIME results for the NNET and XGBoost models show clear differences in feature influence and interpretability. In the NNET model, predictions for the *X2* label are strongly influenced by demographics such as *Family_mem_with_ASD*, *Ethnicity*, and *Rater*, but explanation fits are generally lower, indicating moderate interpretability and some ambiguity in understanding the model’s decision process. For *X1* predictions, mixed positive and negative contributions from traits such as *Gender* and *A2* add complexity to interpretation. In contrast, the XGBoost model’s LIME results for label *X2* consistently show high explanation fits (around 0.7), with top traits such as *A5*, *A6*, and *A4* providing strong support, making these explanations more interpretable and consistent with the model’s decisions. However, for label *X1* predictions, XGBoost has lower explanation fits (around 0.15), indicating less agreement with the model’s decision and a more difficult interpretation, similar to NNET’s *X1* cases. In summary, while both models highlight key features for each label, XGBoost generally provides clearer explanations with higher fits for label *X2*, while NNET has more variation and lower overall interpretability.

**Figure 7:**
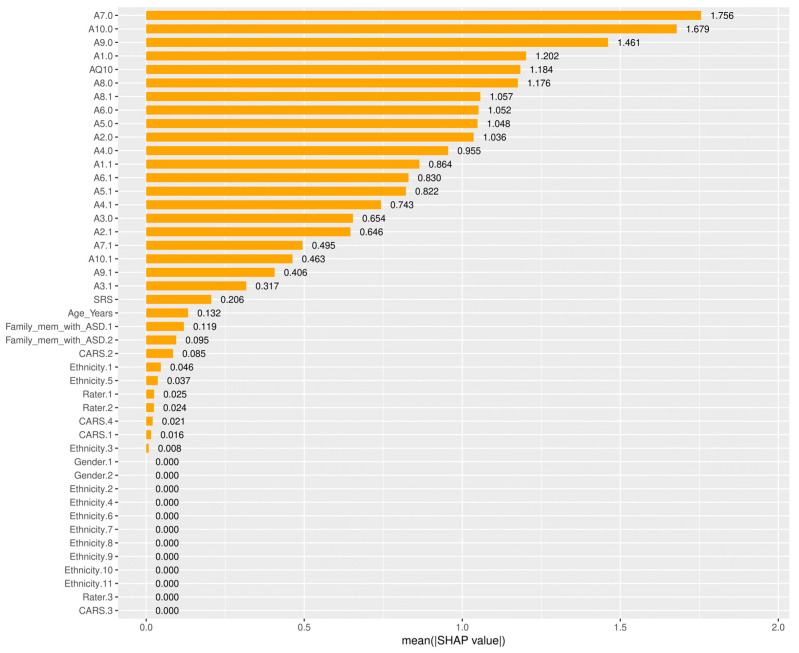
Shapley additive explanation (SHAP) absolute value bar plot. The *x*-axis represents the mean absolute SHAP value for each feature, showing how much each feature contributes to the model’s predictions on average. The *y*-axis lists the features in descending order of importance, with the most important features at the top.

**Figure 8:**
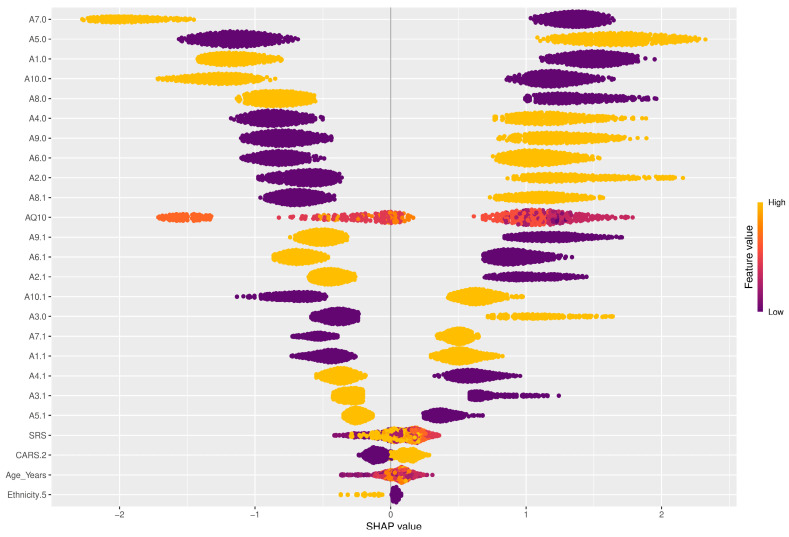
SHAP bee-swarm plot. The *x*-axis represents the SHAP value, which indicates the direction and magnitude of each feature’s effect on the prediction. The *y*-axis lists the features in order of importance, with the most influential features at the top. The color of the dots represents the value of the feature, from low (purple) to high (yellow).

**Figure 9:**
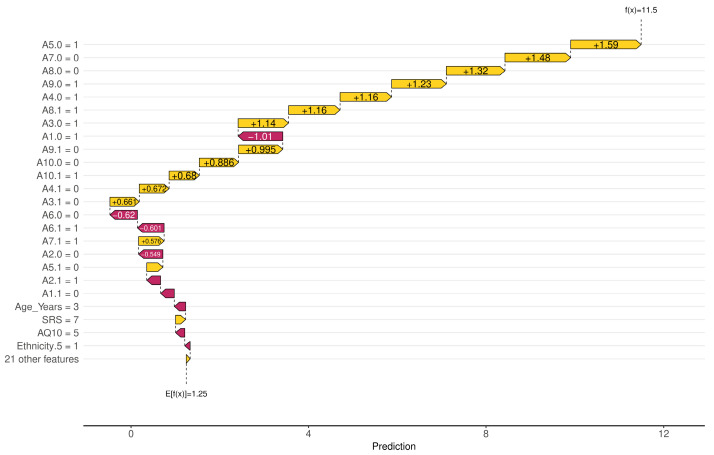
SHAP waterfall plot. The *x*-axis represents the cumulative contribution of each feature to the model’s prediction, starting from the base value to the final prediction. The *y*-axis lists the features in descending order based on their contribution to the prediction for a given instance.

**Figure 10:**
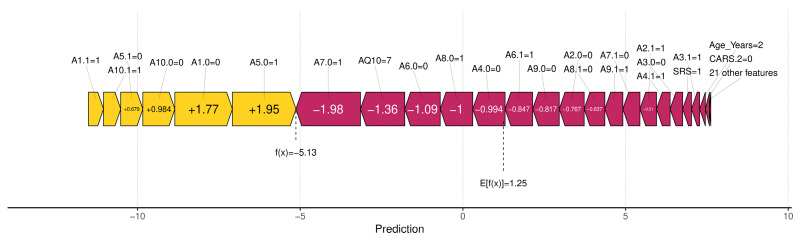
SHAP force plot. The *x*-axis represents the model’s prediction, showing how different features contribute to the final predicted value. The *y*-axis is not explicitly labeled, but the plot visually shows the positive and negative contributions of each feature, represented by the color and direction of the forces.

Among the SHAP packages provided by *R*, *shapviz* [76] is notable for visualizing SHAP values, but it is limited to certain models, such as XGBoost and LightGBM. We applied the SHAP technique using the XGBoost model available in the built-in *caret* library. The visualization results of SHAP are shown in Figure 7, Figure 8, Figure 9 and Figure 10. The SHAP summary plot, shown in Figure 7, highlights *A7.0*, *A10.0*, and *A9.0* as having the strongest positive effects on XGBoost predictions, while variables such as gender and ethnicity had minor effects. The SHAP swarm plot, shown in Figure 8, visually summarizes the feature contributions across data points. Positive SHAP values increase predictions, while negative values decrease predictions. The features at the top, such as *A7.0*, *A5.0*, and *A1.0*, significantly affect predictions, with *A7.0* typically decreasing and *A5.0* typically increasing the predicted value.

The SHAP waterfall plot in Figure 9 shows the sequential feature contributions. For example, features *A5.0* = 1, *A7.0* = 0, and *A8.0* = 0 positively influenced the predicted value to 11.5, while features *A1.0* = 1 and *A6.0* = 0 had a negative influence. The SHAP force plot shown in Figure 10 illustrates the predicted value of the model along the *x*-axis, with the feature contributions divided into positive or negative effects on the final predicted value (*f*(*x*)). *E*[*f*(*x*)] is the standard predicted value, the average value when the model is predicted without features. The final predicted value of XGBoost is −5.13, influenced by features that either increase or decrease this value. Starting with a default of 1.25, features such as *A1.1* = 1 and *A1.0* = 0 increase the predicted value, while *A7.0* = 1 and *AQ10* = 7 decrease it to −5.13.

When comparing PFI, LIME, and SHAP, we see that PFI provides a broad, global understanding of the meaning of features throughout the model. However, it lacks the detailed, instance-specific explanations that SHAP provides. SHAP values provide a more granular interpretation that clearly shows the contribution of each feature to each prediction, allowing for both local and global interpretability. While LIME is useful for generating localized explanations for individual predictions, it lacks the consistency and additive properties of SHAP scores. SHAP’s ability to explain both local and global behavior, while maintaining consistency with the model’s overall predictions, makes it more reliable than LIME for understanding both feature importance and decision-making processes.

By integrating SHAP, LIME, and PFI, this study provides a comprehensive analysis of the inner workings of ML models. These techniques improve both model accuracy and interpretability, providing critical insights into the most influential features for predicting ASD outcomes. Among them, SHAP stands out for providing more comprehensive and reliable explanations compared to PFI and LIME. It excels at explaining both global and local model behavior, making it the preferred choice for understanding complex decision-making processes in the context of ASD prediction. For non-experts, this means that we can not only trust the predictions but also understand how these predictions are made, ensuring transparency in AI-driven healthcare.

## 5. Discussion

Because of the exceptionally high-performance metrics observed in our initial model evaluations, we were concerned about the potential for overfitting. To thoroughly investigate this possibility, we conducted additional cross-validation experiments using 2-fold, 4-fold, and 5-fold cross-validation, as detailed in Table 9, Table 10 and Table 11. These additional experiments were essential to ensure that the excellent performance of models such as NNET, XGBoost, and GBM was not simply a result of overfitting to the specific dataset used in this study.

Table 9 shows the results of the 2-fold cross-validation. XGBoost achieved an impressive accuracy of 0.9968, an F1 score of 0.9978, and perfect precision and recall values of 1 and 0.9956, respectively. GBM also performed exceptionally well, with an accuracy of 0.9889 and an F1 score of 0.9924. In contrast, the NNET model showed an accuracy of 0.8691, indicating variability in performance depending on the cross-validation fold.

Table 10 details the results of the 4-fold cross-validation. Here, XGBoost maintained a high accuracy of 0.9952 and an F1 score of 0.9967, while GBM reached an accuracy of 0.9950 and an F1 score of 0.9967. Notably, the NNET model achieved perfect performance metrics (accuracy = 1; F1 score = 1) in this setting, raising concerns about potential overfitting to the specific data characteristics present in this fold.

Table 11 shows the performance metrics for a 5-fold cross-validation. XGBoost continued to show a strong performance, with an accuracy of 0.9968 and an F1 score of 0.9978. GBM continued to improve, with an accuracy of 0.9984 and an F1 score of 0.9989. The NNET model again showed perfect performance (accuracy = 1; F1 score = 1), consistent with the 4-fold results.

These consistent performance metrics across different cross-validation approaches suggest that XGBoost, NNETs, and GBM have some degree of generalizability, maintaining a stable performance despite variations in training and testing splits. However, the exceptionally high performance of the NNET model in certain folds, achieving perfect scores, indicates a potential risk of overfitting to specific data characteristics within those folds. This concern is further supported by the identical performance metrics observed in some cases for the NNET and *k*-NN models, as shown in Table 7 and Table 8, which may indicate redundancy or overfitting in the model implementation or data processing.

The XGBoost and GBM models displayed a robust predictive performance, with high prAUC values, yet it is essential to acknowledge that their success may still be influenced by unique features of the dataset used in this study. To ensure that these models perform reliably in diverse real-world scenarios, future research should include validation on independent datasets with different demographic and clinical characteristics. This additional validation will help confirm the generalizability and reliability of the models outside of the specific dataset used in this study.

While this study demonstrates the effectiveness of ML models in diagnosing ASD, several limitations should be acknowledged. First, despite the extensive nature of our dataset, it may not be representative of all populations, as ASD symptoms vary widely across demographic groups. A larger, more diverse dataset would likely improve the generalizability and applicability of the models to broader clinical settings. Another important limitation concerns class imbalance: although we used prAUC to handle imbalanced data, models such as *k*-NN and C5.0 struggled to effectively discriminate between ASD and non-ASD cases. Future studies could employ techniques such as synthetic minority oversampling technique (SMOTE) or cost-sensitive learning to further improve performance with unbalanced data.

Moreover, while our study focused on using *R* due to its accessibility and widespread use in the medical field, we did not use the advanced deep-learning frameworks available in *Python*, such as *TensorFlow*, *Keras*, or *PyTorch*. This decision was primarily influenced by our emphasis on tabular datasets rather than image-based data, such as MRI scans, which were not available in our dataset. Complex models such as NNET and XGBoost achieved high accuracy but posed interpretability challenges even with XAI techniques. To mitigate this, we used SHAP and LIME, which provided valuable insights; however, simpler models such as logistic regression may be preferred where interpretability is critical, even at the expense of some accuracy.

Furthermore, we recognize the need for further discussion on the practical adoption and use of these tools, including the development of user-friendly interfaces and addressing potential clinician or caregiver resistance. This would facilitate a smoother integration of AI-based diagnostic tools into clinical practice. Integrating AI-driven diagnostic tools into clinical practice presents significant challenges beyond model performance, requiring a focus on user-friendly interface design and extensive clinician training. Developing intuitive interfaces ensures that healthcare professionals can easily navigate and interpret model output without requiring extensive technical expertise.

Overcoming clinician resistance requires demonstrating the practical utility and reliability of AI tools through evidence-based validation and aligning model predictions with established clinical guidelines. While XAI techniques such as SHAP and LIME increase model transparency by explaining feature contributions, these explanations must also be clinician-centric, translating complex model insights into actionable clinical knowledge. Balancing the computational complexity of advanced models with the need for real-time applicability in busy clinical environments is essential.

Finally, our current reliance on *R* limited our ability to implement more sophisticated deep-learning models that *Python*’s libraries facilitate, particularly those that could handle multimodal data integration. Therefore, future efforts should prioritize the creation of seamless integration workflows to ensure that AI tools complement, rather than disrupt, existing clinical processes. In addition, fostering collaboration between data scientists and clinicians can facilitate the development of models that are both technically robust and clinically relevant, ultimately promoting the widespread adoption and effective use of AI-driven diagnostic tools in healthcare settings.

Future research directions should emphasize the integration of multimodal data, including genetic, neuroimaging, and clinical data, to enrich datasets and provide a comprehensive view of ASD in children, thereby improving both diagnostic accuracy and personalized care. Incorporating imaging data, such as MRI scans, would likely improve the performance of both ML and DNN models by providing valuable structural and functional information about the brain. However, our current study did not include such data and instead focused on survey-based assessments. Another focus could be the real-time implementation of models in clinical settings, especially for childcare, which requires the development of user-friendly interfaces and mobile applications for healthcare professionals. Addressing class imbalance in rare conditions such as ASD through advanced methods such as SMOTE and ensemble learning could lead to more balanced and accurate results during model training. In addition, improving the interpretability of AI models for non-experts, such as parents and caregivers, is essential. Simplified AI output and visual aids can help these stakeholders understand and effectively use advanced models to support their child’s care.

Future research should prioritize the development of AI-driven systems that facilitate early, personalized interventions for children with ASD. While *R* was used in this study due to its accessibility and widespread use in the medical field, we intend to explore *Python* in future studies to take advantage of its extensive libraries and community support, such as *TensorFlow*, *Keras*, and *PyTorch* for deep learning, and to incorporate a wider range of data, including MRI images [77,78], voice recordings, and video analysis. Additionally, we plan to integrate genetic expression data [79] and gut microbiome data [80]. This transition will enable the use of more advanced machine-learning and deep neural-network models, thereby expanding the scope and applicability of our research in clinical settings. While *R* was used in this study due to its accessibility and widespread use in the medical field, we are committed to exploring *Python* in future research to take advantage of its extensive libraries and community support. This transition will enable the use of more advanced machine-learning libraries and foster greater collaboration within the childcare research community.

## 6. Conclusions

This study was guided by four primary research questions, which we addressed through a comprehensive ML framework using *R* and the *caret* package.

- First, to evaluate the impact of a rigorous data-preprocessing pipeline—including outlier removal, missing data handling, and expert-driven feature selection—we meticulously implemented these steps to improve the performance, robustness, and generalizability of ML models for ASD diagnosis. This approach was particularly critical in the context of data heterogeneity and imbalance, ensuring that the models could effectively handle diverse and imbalanced datasets.
- Second, we compared different ML algorithms (e.g., SVMs, RFs, XGBoost, and NNETs) in terms of accuracy, interpretability, and computational efficiency, using *R*. The results highlighted trade-offs between model complexity and practical usability in clinical settings. For example, while complex models such as NNET and XGBoost showed superior accuracy, simpler models such as logistic regression offered greater interpretability, which is essential for clinical decision-making.
- Third, we integrated advanced XAI techniques (e.g., PFI, LIME, and SHAP) to improve the interpretability of ML models for ASD diagnosis. These methods revealed that some features traditionally emphasized in clinical assessments, such as *CARS*, *SRS*, and *AQ10*, were less significant in the ML models, likely due to data imbalance, with a higher number of ASD patient interviews. This finding helps to improve communication between clinicians and AI models, promoting trust and usability.
- Finally, we developed accessible ML tools using *R* and the *caret* package to facilitate the adoption of AI-driven diagnostic methods by clinicians. By leveraging *R*’s accessibility and widespread use in the medical field, we aimed to ensure that these tools could be used effectively without requiring extensive programming expertise, thereby improving the effectiveness and reliability of ASD diagnosis in clinical practice.

A major contribution of this study is the use of XAI techniques, which provided clearer insights into the decision-making processes of the models. These techniques revealed that specific behavioral characteristics, such as social communication patterns, were more important than demographic factors or family history in diagnosing ASD in children. This finding is consistent with the multifaceted nature of ASD, where individual behavioral indicators in children are critical. By increasing transparency, XAI enabled clinicians to better understand the models’ predictions, thereby increasing confidence in AI-based diagnostic tools. This study demonstrates the potential of ML models to improve early diagnosis of ASD in children by emphasizing both interpretability and accuracy.

Moving forward, it is imperative to validate these models in diverse clinical settings, particularly pediatric-care settings, to ensure robustness and generalizability. In addition, the exploration of advanced regularization techniques and the use of cross-validation methods will be critical to further improve accuracy and reduce the risk of overfitting. By refining these models, we can significantly improve diagnostic tools for ASD in children, leading to more effective intervention planning and improved quality of life for children and their families. These efforts will not only advance healthcare analytics but also foster greater trust in AI applications in childcare, contributing to emotionally intelligent, child-focused clinical practices.

## Figures and Tables

**Table 1:** Comparison between previous studies on autism spectrum disorder (ASD) diagnosis and intervention and the present study.

Authors	Year	Contributions	Differences from Present Study
Lord and Luyster [5]	2006	Demonstrated stability of ASD diagnosis from age 2 to 9; emphasized reliability of early diagnosis.	Does not incorporate ML or XAI; focuses on longitudinal stability rather than predictive modeling.
McCarty and Frye [6]	2020	Identified challenges in early diagnosis; proposed multi-stage screening to improve diagnostic accuracy.	Does not utilize ML algorithms or XAI techniques; focuses on screening methodologies.
Bryson et al. [7]	2003	Reviewed impact of early intervention on developmental outcomes; emphasized importance of early detection tools.	Lacks application of ML models and XAI; focuses on intervention strategies rather than predictive analytics.
Guthrie et al. [8]	2013	Examined stability of early diagnoses; highlighted need for multifaceted diagnostic approaches.	Does not apply ML or XAI; emphasizes clinical expertise and multi-source information integration.
Omar et al. [12]	2019	Developed ML models using RF-CART and RF-ID3; created a mobile diagnostic application for ASD.	Focuses on model development and application without integrating XAI techniques; limited interpretability of model predictions.
Usta et al. [13]	2019	Evaluated ML algorithms for predicting short-term ASD outcomes; found decision tree to be most effective.	Does not incorporate XAI for model interpretability; primarily focuses on prognosis prediction rather than diagnostic accuracy.
Alsuliman and Al-Baity [26]	2022	Developed optimized ML models for ASD classification using PBC and GE data with bio-inspired algorithms.	Does not integrate advanced XAI techniques to improve both the accuracy and interpretability of their models.
Ben-Sasson et al. [27]	2023	Developed a gradient boosting model for early ASD prediction using electronic health records.	Lacks integration of XAI techniques; focuses on predictive modeling using electronic health records.
Abbas et al. [28]	2023	Compared TPOT and KNIME for ASD detection, focusing on feature selection.	Does not include XAI techniques; focuses on comparing AutoML tools for ASD detection.
Reghunathan et al. [29]	2023	Used machine-learning classifiers for ASD detection, with logistic regression showing the highest accuracy.	Does not use XAI techniques; focuses on feature reduction and classifier accuracy.
Bala et al. [30]	2023	Built an ASD detection model across age groups, with SVM performing best.	Focuses on model performance across age groups without applying XAI for interpretability.
Batsakis et al. [31]	2023	Built a data-driven AI model for clinical ASD diagnosis, highlighting data limitations.	Emphasizes model development using AutoML without integrating XAI for improved interpretability.
Our Study	2024	Developed interpretable ML models using XAI techniques; implemented rigorous data preprocessing; provided guidelines for non-experts.	Integrates XAI for model transparency; emphasizes data reliability and preprocessing; offers practical guidelines for clinical use.

**Table 2:** Detailed overview of ASD children trait dataset with variable characteristics.

Variable	Description	Range/Values	Missing Data
CASE_NO_PATIENT’S	Unique identifier for each patient case	1 to 1985	No
A1–A10	Behavioral indicators, measured as binary values, reflecting certain autistic traits	0 or 1	No
Social_Responsiveness_Scale	Score measuring social responsiveness; higher values indicate more difficulties	0 to 10	Yes
Age_Years	The age of the child in years	1 to 18	No
Qchat_10_Score	Questionnaire score assessing autistic traits in young children	0 to 10	Yes
Speech Delay/Language Disorder	Whether the child has speech or language delays	Yes, no	No
Learning Disorder	Presence of learning disabilities	Yes, no	No
Genetic Disorders	Whether the child has any known genetic disorders	Yes, no	No
Depression	Indicates if the child has depression	Yes, no	Yes
Global Developmental Delay/Intellectual Disability	Indicates the presence of developmental delays or intellectual disabilities	Yes, no	No
Social/Behavioral Issues	Whether the child exhibits social or behavioral problems	Yes, no	Yes
Childhood Autism Rating Scale (CARS)	A clinical tool to rate autism severity (1 = nothing, 2 = little, 3 = medium, and 4 = severe)	1 to 4	No
Anxiety Disorder	Indicates if the child has been diagnosed with anxiety disorders	Yes, no	No
Sex	Gender of the child	M, F	No
Ethnicity	Ethnic background of the child	Asian, Black, Hispanic, Latino, Middle Eastern, Mixed, Native Indian, PaciFica, South Asian, White European, others	No
Jaundice	Whether the child had jaundice at birth	Yes, no	No
Family_mem_with_ASD	Indicates if a family member has been diagnosed with ASD	Yes, no	No
Who_completed_the_test	The person who completed the assessment	Family member, Healthcare professional, others	No
ASD_traits (dependent variable)	Final diagnostic classification for ASD	Yes, no	No

**Table 3:** Preprocessed dataset characteristics.

Variable	Description
Gender	Categorical variable: 1 = boy; 2 = girl.
Age_Years	Numeric variable. Represents the age of the child (range: 1–18 years).
Ethnicity	Categorical variable: 1 = Asian, 2 = Black, 3 = Hispanic, 4 = Latino, 5 = Middle Eastern, 6 = Mixed, 7 = Native Indian, 8 = Others, 9 = Pacifica, 10 = South Asian, and 11 = White European.
Family_mem_with_ASD	Binary variable: 1 = yes; 2 = no. Indicates if any family member has ASD.
Rater	Categorical variable indicating who completed the test. 1 = Family member, 2 = Healthcare professional, 3 = Others.
ASD_traits (Dependent Variable)	Binary variable: 1 = yes; 2 = no. Represents if ASD traits are present.
Social Responsiveness Scale (SRS)	Numeric variable ranging from 1 to 10. Measures the severity of social impairment. Missing values are present.
CARS	Numeric variable ranging from 1 to 10. Higher values indicate more severe symptoms. Missing values are present.
A1 to A10 (Autism Spectrum Quotient)	Binary variables (0 = no; 1 = yes). Represents responses to a series of questions related to autism traits.
AQ10 (Autism Quotient Score)	Numeric variable ranging from 1 to 10. Measures autism-trait severity.

**Table 4:** Performance metrics for each model based on 10-fold cross-validation.

Model	Accuracy	F1 Score	prAUC	Precision	Recall
Random forest	0.9463	0.9642	0.9317	0.9409	0.9891
SVM	0.9874	0.9913	0.9545	0.9892	0.9934
GBM	0.9984	0.9989	0.9601	1	0.9978
XGBoost	0.9984	0.9989	0.9103	1	0.9978
C5.0	0.9701	0.9796	0.5358	0.9705	0.9892
NNET	1	1	0.9601	1	1
*k*-NN	0.8883	0.9231	0.5984	0.9156	0.9331
Logistic regression	0.9858	0.9903	0.9576	0.9873	0.9935

**Table 5:** Wilcoxon signed-rank test results (*p*-values) with NNET with other models based on fold-wise performance metrics.

Model	Accuracy	F1 Score	prAUC	Precision	Recall
Random forest	0.003	0.003	0.001	0.027	0.003
SVM	0.049	0.049	0.140	0.500	0.087
GBM	0.500	0.500	0.047	0.500	1.000
XGBoost	0.500	0.500	0.007	1.000	0.500
C5.0	0.007	0.007	0.001	0.024	0.007
*k*-NN	0.003	0.001	0.001	0.003	0.001
Logistic regression	0.091	0.087	0.248	0.500	0.186

**Table 6:** Friedman test results across all models based on fold-wise performance metrics.

Metric	Chi Squared	df	*p*-Value
Accuracy	56.161	7	8.775 × 10^−10^
F1 Score	57.058	7	5.822 × 10^−10^
prAUC	57.416	7	4.942 × 10^−10^
Precision	42.265	7	4.623 × 10^−7^
Recall	57.377	7	5.030 × 10^−10^

**Table 7:** Optimization results for neural network (NNET) hyperparameters.

Size	Decay	Accuracy	F1 Score	prAUC	Precision	Recall
5	0.01	1	1	0.9600	1	1
5	0.1	1	1	0.9601	1	1
7	0.01	1	1	0.9524	1	1
7	0.1	1	1	0.9600	1	1
10	0.01	1	1	0.9600	1	1
10	0.1	1	1	0.9600	1	1
15	0.01	1	1	0.8900	1	1
15	0.1	1	1	0.9600	1	1
20	0.01	1	1	0.7354	1	1
20	0.1	1	1	0.9526	1	1

**Table 8:** Detailed evaluation of *k*-NN performance across different *k*-values.

k	Accuracy	F1 Score	prAUC	Precision	Recall
1	0.8380	0.8802	0.1145	0.8971	0.8672
2	0.8284	0.8749	0.1765	0.8829	0.8695
3	0.8680	0.9024	0.3057	0.9092	0.8977
4	0.8517	0.8890	0.3815	0.8994	0.8998
5	0.8695	0.9049	0.4465	0.8987	0.9127
6	0.8618	0.8983	0.4770	0.8976	0.9019
7	0.8883	0.9231	0.5984	0.9156	0.9331
8	0.8670	0.8969	0.5573	0.8905	0.9150
9	0.8601	0.8894	0.5818	0.8807	0.8993
10	0.8602	0.8893	0.6005	0.8834	0.9071

**Table 9:** Model performance metrics using 2-fold cross-validation.

Model	Accuracy	F1 Score	prAUC	Precision	Recall
Random forest	0.9463	0.9642	0.9665	0.9408	0.9891
SVM	0.9605	0.9729	0.9832	0.9739	0.9718
GBM	0.9889	0.9924	0.9900	0.9913	0.9935
XGBoost	0.9968	0.9978	0.9871	1	0.9956
C5.0	0.9621	0.9741	0.7893	0.9679	0.9805
NNET	0.8691	0.9109	0.4091	0.9023	0.9199
*k*-NN	0.8691	0.9109	0.4091	0.9023	0.9199
Logistic regression	0.9684	0.9785	0.9882	0.9702	0.9870

**Table 10:** Model performance metrics using 4-fold cross-validation.

Model	Accuracy	F1 Score	prAUC	Precision	Recall
Random forest	0.9431	0.9623	0.9483	0.9374	0.9891
SVM	0.9842	0.9891	0.9821	0.9912	0.9870
GBM	0.9950	0.9967	0.9838	0.9957	0.9978
XGBoost	0.9952	0.9967	0.9506	0.9957	0.9978
C5.0	0.9526	0.8678	0.7735	0.9556	0.9804
NNET	1	1	0.9245	1	1
*k*-NN	0.8927	0.9273	0.6730	0.9157	0.9393
Logistic regression	0.9889	0.9924	0.9828	0.9914	0.9935

**Table 11:** Model performance metrics using 5-fold cross-validation.

Model	Accuracy	F1 Score	prAUC	Precision	Recall
Random forest	0.9385	0.9586	0.9353	0.9380	0.9804
SVM	0.9889	0.9923	0.9782	0.9934	0.9913
GBM	0.9984	0.9989	0.9800	1	0.9978
XGBoost	0.9968	0.9978	0.9700	0.9978	0.9870
C5.0	0.9763	0.9838	0.6667	0.9800	0.9870
NNET	1	1	0.9216	1	1
*k*-NN	0.8896	0.9259	0.6847	0.9100	0.9436
Logistic regression	0.9921	0.9946	0.9791	0.9914	0.9978

## Data Availability

The dataset utilized in this study, titled ‘ASD Children Traits’, is publicly accessible and can be obtained from Kaggle at the following URL: https://www.kaggle.com/datasets/uppulurimadhuri/dataset (accessed on 1 June 2024). Additional data supporting the findings of this research are available from the corresponding author upon reasonable request.

## Supplementary Materials

The following supporting information can be downloaded at: https://www.mdpi.com/article/10.3390/diagnostics14222504/s1, Table S1: ASD_Preprocessed_Dataset.xlsx, which is the preprocessed dataset used for our experiments, including brief descriptions of each variable’s values and characteristics.

## Appendix A

The following tables present the detailed performance metrics for each cross-validation fold used in the evaluation of multiple ML models for diagnosing ASD. These metrics complement the main text and provide comprehensive insights necessary to understand and replicate the research. In addition, these detailed results facilitate the application of statistical tests to assess the significance of performance differences between models.

**Table A1:** Performance metrics for fold 1.

Model	Accuracy	F1 Score	prAUC	Precision	Recall
Random forest	0.9523	0.9677	0.9405	0.9782	0.9574
SVM	0.9687	0.9791	0.9493	1	0.9591
GBM	1	1	0.9597	1	1
XGBoost	1	1	0.9287	1	1
C5.0	1	1	0.6323	1	1
NNET	1	1	0.9599	1	1
*k*-NN	0.8281	0.8705	0.7232	0.8043	0.9487
Logistic regression	1	1	0.9597	1	1

**Table A2:** Performance metrics for fold 2.

Model	Accuracy	F1 Score	prAUC	Precision	Recall
Random forest	0.9523	0.9684	0.9271	1	0.9387
SVM	1	1	0.9599	1	1
GBM	0.9841	0.9890	0.9557	0.9782	1
XGBoost	0.9682	0.9787	0.9340	1	0.9583
C5.0	0.9687	0.9782	0.5509	0.9782	0.9782
NNET	1	1	0.9597	1	1
*k*-NN	0.8095	0.8750	0.5080	0.9130	0.8400
Logistic regression	0.9841	0.9892	0.9578	1	0.9787

**Table A3:** Performance metrics for fold 3.

Model	Accuracy	F1 Score	prAUC	Precision	Recall
Random forest	0.9687	0.9791	0.9117	1	0.9591
SVM	1	1	0.9613	1	1
GBM	1	1	0.9613	1	1
XGBoost	1	1	0.9599	1	1
C5.0	0.9843	0.9892	0.7222	1	0.9787
NNET	1	1	0.9613	1	1
*k*-NN	0.9375	0.9583	0.6450	0.9787	0.9387
Logistic regression	1	1	0.9597	1	1

**Table A4:** Performance metrics for fold 4.

Model	Accuracy	F1 Score	prAUC	Precision	Recall
Random forest	0.9523	0.9684	0.9587	1	0.9387
SVM	0.9841	0.9892	0.9597	1	0.9787
GBM	1	1	0.9597	1	1
XGBoost	1	1	0.9597	1	1
C5.0	0.9682	0.9782	0.6953	0.9782	0.9782
NNET	1	1	0.9597	1	1
*k*-NN	0.8730	0.9111	0.6264	0.8913	0.9318
Logistic regression	1	1	0.9597	1	1

**Table A5:** Performance metrics for fold 5.

Model	Accuracy	F1 Score	prAUC	Precision	Recall
Random forest	0.9375	0.9583	0.9291	0.9787	0.9387
SVM	1	1	0.9597	1	1
GBM	1	1	0.9194	1	1
XGBoost	1	1	0.9173	1	1
C5.0	0.9523	0.9648	0.6030	1	0.9387
NNET	1	1	0.9615	1	1
*k*-NN	0.9206	0.9462	0.6924	0.9565	0.9361
Logistic regression	0.9843	0.9892	0.9560	0.9787	1

**Table A6:** Performance metrics for fold 6.

Model	Accuracy	F1 Score	prAUC	Precision	Recall
Random forest	0.9047	0.9361	0.8983	0.9565	0.9166
SVM	1	1	0.9597	1	1
GBM	1	1	0.9597	1	1
XGBoost	1	1	0.9271	1	1
C5.0	0.9531	0.9684	0.6547	0.9787	0.9583
NNET	1	1	0.9597	1	1
*k*-NN	0.9206	0.9450	0.6106	0.9347	0.9555
Logistic regression	1	1	0.9599	1	1

**Table A7:** Performance metrics for fold 7.

Model	Accuracy	F1 Score	prAUC	Precision	Recall
Random forest	0.9682	0.9787	0.9162	1	0.9583
SVM	0.9841	0.9892	0.9560	1	0.9787
GBM	1	1	0.9198	1	1
XGBoost	1	1	0.9597	1	1
C5.0	1	1	0.4561	1	1
NNET	1	1	0.9597	1	1
*k*-NN	0.9062	0.9361	0.6021	0.9565	0.9166
Logistic regression	1	1	0.9613	1	1

**Table A8:** Performance metrics for fold 8.

Model	Accuracy	F1 Score	prAUC	Precision	Recall
Random forest	0.9365	0.9574	0.9201	0.9782	0.9375
SVM	1	1	0.9597	1	1
GBM	1	1	0.9613	1	1
XGBoost	1	1	0.9488	1	1
C5.0	0.9682	0.9782	0.6529	0.9782	0.9782
NNET	1	1	0.9597	1	1
*k*-NN	0.9206	0.9473	0.6730	0.9782	0.9183
Logistic regression	1	1	0.9597	1	1

**Table A9:** Performance metrics for fold 9.

Model	Accuracy	F1 Score	prAUC	Precision	Recall
Random forest	0.9687	0.9787	0.9437	1	0.9583
SVM	1	1	0.9597	1	1
GBM	1	1	0.9305	1	1
XGBoost	1	1	0.8836	1	1
C5.0	0.9682	0.9787	0.7966	1	0.9583
NNET	1	1	0.9597	1	1
*k*-NN	0.9365	0.9565	0.5708	0.9565	0.9565
Logistic regression	0.9687	0.9787	0.9613	1	0.9583

**Table A10:** Performance metrics for fold 10.

Model	Accuracy	F1 Score	prAUC	Precision	Recall
Random forest	0.9531	0.9677	0.9015	0.9782	0.9574
SVM	0.9843	0.9890	0.9613	0.9782	1
GBM	1	1	0.9597	1	1
XGBoost	1	1	0.9178	1	1
C5.0	0.9365	0.9574	0.6453	0.9782	0.9375
NNET	1	1	0.9597	1	1
*k*-NN	0.8750	0.9200	0.5928	0.9787	0.8679
Logistic regression	1	1	0.9597	1	1
